# Potential Pathogenicity Determinants Identified from Structural Proteomics of SARS-CoV and SARS-CoV-2

**DOI:** 10.1093/molbev/msaa231

**Published:** 2020-09-17

**Authors:** Erica T Prates, Michael R Garvin, Mirko Pavicic, Piet Jones, Manesh Shah, Omar Demerdash, B Kirtley Amos, Armin Geiger, Daniel Jacobson

**Affiliations:** 1 Biosciences Division, Oak Ridge National Laboratory, Oak Ridge, TN; 2 National Virtual Biotechnology Laboratory, US Department of Energy, TN; 3 The Bredesen Center for Interdisciplinary Research and Graduate Education, The University of Tennessee Knoxville, Knoxville, TN; 4 Genome Science and Technology, The University of Tennessee Knoxville, Knoxville, TN; 5 Department of Horticulture, N-318 Ag Sciences Center, University of Kentucky, Lexington, KY; 6 Department of Psychology, The University of Tennessee Knoxville, Knoxville, TN

**Keywords:** COVID-19, SARS-CoV-2, SARS-CoV, pathogenesis, proteome-wide analysis, proteome-wide modeling

## Abstract

Despite SARS-CoV and SARS-CoV-2 being equipped with highly similar protein arsenals, the corresponding zoonoses have spread among humans at extremely different rates. The specific characteristics of these viruses that led to such distinct outcomes remain unclear. Here, we apply proteome-wide comparative structural analysis aiming to identify the unique molecular elements in the SARS-CoV-2 proteome that may explain the differing consequences. By combining protein modeling and molecular dynamics simulations, we suggest nonconservative substitutions in functional regions of the spike glycoprotein (S), nsp1, and nsp3 that are contributing to differences in virulence. Particularly, we explain why the substitutions at the receptor-binding domain of S affect the structure–dynamics behavior in complexes with putative host receptors. Conservation of functional protein regions within the two taxa is also noteworthy. We suggest that the highly conserved main protease, nsp5, of SARS-CoV and SARS-CoV-2 is part of their mechanism of circumventing the host interferon antiviral response. Overall, most substitutions occur on the protein surfaces and may be modulating their antigenic properties and interactions with other macromolecules. Our results imply that the striking difference in the pervasiveness of SARS-CoV-2 and SARS-CoV among humans seems to significantly derive from molecular features that modulate the efficiency of viral particles in entering the host cells and blocking the host immune response.

## Introduction

Global infections from SARS-CoV-2, the betacoronavirus that causes COVID-19, recently surpassed 29 million. In contrast, SARS-CoV, the most closely related zoonotic virus to SARS-CoV-2 (Coronaviridae Study Group of the International Committee on Taxonomy of Viruses 2020), sharing nearly 80% sequence identity, had a much more limited geographic distribution, with around 8,000 cases reported (WHO 2003). Both viruses use their spike glycoprotein (S) to co-opt the protease angiotensin-converting enzyme 2 (ACE2) to enter host cells, as does the related alphacoronavirus HCoV-NL63, which has been reported in several countries, but with rare mortality (Abdul-Rasool and Fielding 2010). Multiple sociodemographic factors contribute to the magnitude of the pervasiveness and mortality rates of these viruses, but differences in viral proteomes are quite likely to affect viral pathobiology (Andersen et al. 2020; Wilder-Smith et al. 2020; Xu et al. 2020). Although individuals infected with SARS-CoV and SARS-CoV-2 present similar primary symptoms, the COVID-19 pandemic has clearly demonstrated that SARS-CoV-2 evolved different strategies that allowed for a more efficient and rapid spread, but genomic changes and molecular processes that underlie this remain unclear.

Structural analyses of phylogenetically related viruses can provide a better understanding of the key molecular features determining different pathotypes. For example, an earlier study demonstrated that the specific variation in the membrane-proximal region of the S protein between feline alphacoronaviruses alters the tropism from an intestinally focused infection to the ability of the virus to replicate in macrophages, causing higher mortality rates (Rottier et al. 2005). In a similar line of research, a recent study revealed that SARS-CoV-2 replicates better than other coronaviruses, including SARS-CoV, in the human bronchus, and that may contribute to the higher transmission rate of COVID-19 (Hui et al. 2020). The authors suggest that the insertion of a polybasic motif that is susceptible to proteolysis (Coutard et al. 2020; Walls et al. 2020) at the junction of the S1 and S2 subunits, combined with the high expression of the TMPRSS2 protease that cleaves it in bronchial tissues, may be an important enhancing factor for the better replication of SARS-CoV-2 compared with SARS-CoV. Experiments with SARS-CoV indicate that this may not result from enhanced virion entry, but rather from enhanced cell–cell fusion (Follis et al. 2006).

The spike glycoproteins sequences are highly conserved between SARS-CoV and SARS-CoV-2 (identity of 77%), but several amino acid substitutions are located in the receptor-binding domain (RBD) of these proteins, which has been suggested to be tightly associated with the distinct outcomes of infection by these viruses (Letko et al. 2020; Ou et al. 2020; Walls et al. 2020; Wrapp et al. 2020). Like the polybasic motif insert, functionally relevant molecular differences in regions that do not directly bind to the host receptor have also been identified. Mapping of SARS-CoV-2 S glycosylation reveals amino acid substitutions that determine its specific glycan signatures and exposed epitopes for antibody neutralization (Shajahan et al. 2020; Watanabe et al. 2020). As a primary determinant of pathogenesis, the S protein of coronaviruses has been a major focus of numerous studies in order to understand the molecular mechanism of infection and explore its potential as a target for vaccines and antiviral treatments. However, several other proteins exhibit unique features in SARS-CoV-2 (Wu et al. 2020), and their functional consequences are currently unknown.

In the present study, we use a structural analysis approach to explore the full viral machinery of SARS-CoV-2 in comparison to SARS-CoV in order to identify the molecular elements that may be enhancing the spread of COVID-19 compared with SARS. We used the currently available experimentally solved structures of SARS-CoV-2 proteins and a robust ensemble workflow to predict structural models with the highest possible resolution for the unsolved proteins. Combined with molecular dynamics (MD) simulations, the analysis of the protein structures suggests specific substitutions within the two proteomes that are likely the major determinants of differences in pathogenicity. We also identify conserved regions that may be promising targets for the development of broad-spectrum antivirals. As part of this report, we provide the scientific community a synopsis and downloadable content of each SARS-CoV-2 proteins with functional insights about the likely impact of mutations on virulence and pathogenicity.

## Results and Discussion

### Overview of the Molecular Differences between SARS-CoV and SARS-CoV-2 Proteomes

**Fig. 1.**
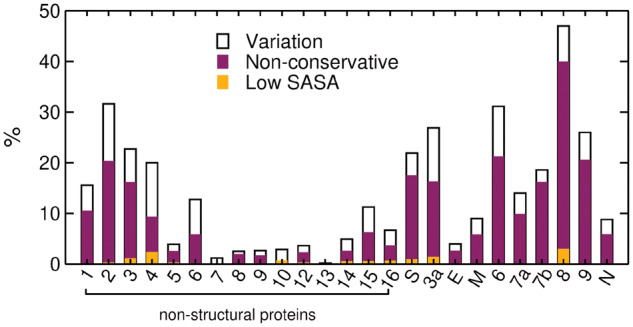
Distribution of sequence variation in fully or partially solved structures of SARS-CoV-2 proteins relative to SARS-CoV. Variations considered nonconservative, represented in *magenta*, are defined in supplementary table S1, Supplementary Material online. Variations occurring within protein cores (low solvent accessible surface area, SASA) are represented in *orange*. ORF10 is not included as it is not found in the SARS-CoV proteome. The percentages were computed relative to the total number of amino acids of each protein.

The proteome of SARS-CoV-2 includes four proteins that constitute the external structure of the virus and the internal framework for storing the RNA genome, namely, S, envelope (E), membrane glycoprotein (M), and nucleocapsid (N). It also produces 16 nonstructural proteins (nsp1–nsp16) and at least seven accessory proteins that function in the replication of the genome, proofreading, proteome processing, and suppression of the host immune response (table 1) (Gordon et al. 2020). An in-depth comparative genome study reported that 380 amino acids that are fixed across thousands of SARS-like coronaviruses are changed, and specific to SARS-CoV-2 (Wu et al. 2020), suggesting that these mutations may be essential for determining the pathogenic divergence of COVID-19. Here, we verified that there are ∼1,570 amino acid substitutions between SARS-CoV-2 and SARS-CoV proteomes, including the 380 highly conserved sites that are specific to SARS-CoV-2 based on a broad evolutionary comparison. As shown in figure 1, the majority of variations are nonconservative and distributed among the mature proteins, whereas several nonstructural proteins are highly similar to their counterparts in SARS-CoV, suggesting strong purifying selection (i.e., protein structure and function are highly conserved and, therefore, mutations in these proteins are selected against). Their likely long-term stability in the population makes them attractive targets for the development of broad-spectrum antivirals as well as good targets for diagnostic primers. Except for ORF8, the most variable sequences diverge ∼30% relative to SARS-CoV, which typically do not change global topologies and, consequently, the main protein function.

**Table 1.** Summary of the SARS-CoV-2 Proteome (reference genome NC_045512.2).

Name	Accession	Length	Function
nsp1	YP_009725297.1	180	Inhibits host gene expression and interferon signaling (Züst et al. 2007; Narayanan et al. 2008, 2015; Kamitani et al. 2009).
nsp2	YP_009725298.1	638	May assist other viral proteins in their function, interacting with several of them, but its specific function is not known yet (Prentice et al. 2004; von Brunn et al. 2007).
nsp3	YP_009725299.1	1,945	Papain-like protease with phosphatase activity. Performs proteolytic cleavage of the polyproteins (Saikatendu et al. 2005). Inhibits components of NF-κB, interferon-beta, and p53 signaling. It may participate in membrane rearrangements with nsp4 (Wathelet et al. 2007; Hagemeijer et al. 2014; Yuan et al. 2015).
nsp4	YP_009725300.1	500	Essential to membrane rearrangements during viral replication (Angelini et al. 2013; Sakai et al. 2017).
nsp5	YP_009725301.1	306	Also known as 3C-like proteinase, its main role is to cleave the viral polyprotein to generate the active forms of the nonstructural proteins (Ziebuhr et al. 2000; Anand et al. 2003; Perlman and Netland 2009).
nsp6	YP_009742613.1	290	Participates in membrane rearrangements and autophagy (Angelini et al. 2013).
nsp7	YP_009725303.1	83	Part of the replication complex (nsp7–nsp8–nsp12). It forms an hexadecameric complex with nsp8 that may act as a processivity clamp for the RNA-dependent RNA polymerase (Zhai et al. 2005; Smith and Denison 2013).
nsp8	YP_009725304.1	198	Part of the replication complex (nsp7–nsp8–nsp12). It forms an hexadecameric complex with nsp7 that may act as a processivity clamp for the RNA-dependent RNA polymerase (Zhai et al. 2005; Smith and Denison 2013).
nsp9	YP_009725305.1	113	Forms homodimers that bind and protect the viral genome from degradation during replication (Sutton et al. 2004; Ponnusamy et al. 2008).
nsp10	YP_009725306.1	139	Forms complexes with nsp14 and nsp16, which perform 3′–5′ exoribonuclease and 2′-*O*-methyltransferase activities, respectively (Bouvet et al. 2010; Wang et al. 2015).
nsp11	YP_009725312.1	13	Short peptide that may be involved in RNA synthesis (Su et al. 2006).
nsp12	YP_009725307.1	932	RNA-dependent RNA polymerase, the core of the replication complex (nsp7–nsp8–nsp12) (Smith and Denison 2013; Gao et al. 2020).
nsp13	YP_009725308.1	601	RNA helicase with NTPase, dNTPase, and RTpase activities (Ivanov and Ziebuhr 2004).
nsp14	YP_009725309.1	527	3′–5′ exonuclease with proofreading activity (Chen et al. 2007; Ma et al. 2015).
nsp15	YP_009725310.1	346	Nidoviral RNA uridylate-specific endoribonuclease (NendoU) (Kim et al. 2020).
nsp16	YP_009725311.1	298	2′-*O*-Ribose methyltransferase. In association with nsp10, it is involved in capping of viral mRNA to protect it from host degradation (Decroly et al. 2011).
S	YP_009724390.1	1,273	Spike glycoprotein. Main means of virus entry into host cells. These highly glycosylated proteins protrude from the viral surface to interact with the host cell receptor(s) (Walls et al. 2020).
M	YP_009724393.1	222	Membrane glycoprotein. Required for membrane curvature initiation, RNA packing, and viral particle budding (Neuman et al. 2011).
N	YP_009724397.2	419	Nucleocapsid. Packages the viral RNA to form a ribonucleocapsid, playing a key role in viral assembly (Chang et al. 2009).
E	YP_009724392.1	75	Envelope protein. Minor structural protein that forms pentameric ion channels in host ER membranes (Li et al. 2014). Involved in overexpression of cytokines and exaggerated immune response (Fang et al. 2007; Siu et al. 2009).
ORF3a	YP_009724391.1	275	Forms homotetramers with ion channel properties (Lu et al. 2006). Linked to inflammatory, IFN and innate immunity responses, it triggers apoptosis and modulates cell cycle (Kanzawa et al. 2006; Yuan et al. 2007; Padhan et al. 2008; Minakshi et al. 2009).
ORF6	YP_009724394.1	61	Enhances viral replication (Huang et al. 2007; Zhao et al. 2009).
ORF7a	YP_009724395.1	121	Prevents virus tethering at the plasma membrane by binding to BTS-2 (Taylor et al. 2015).
ORF7b	YP_009725318.1	43	Integral transmembrane protein. Its function is unclear (Pekosz et al. 2006; Schaecher et al. 2007).
ORF8	YP_009724396.1	121	Accessory protein involved in enhanced virus replication (Muth et al. 2018).
ORF9b^a^	PODTD2		Alternative reading frame in the N gene. Suppresses host antiviral response by promoting MAVS degradation (Shi et al. 2014; Gordon et al. 2020).
ORF10	YP_009725255.1	38	Accessory protein with potential role in inhibiting the ubiquitin-proteasome system (UPS) (Gordon et al. 2020).

a Annotated by Gordon et al. (2020).

Structural knowledge of SARS-CoV proteins is fairly extensive and information about structure–function relationships of SARS-CoV-2 proteins is becoming increasingly available. The visual inspection of nonconserved substitutions in solved and predicted structures combined with analyses of their structural profiles (i.e., predicted location of structured, intrinsically disordered and transmembrane regions—Materials and Methods) indicates that the great majority of them are in surface-exposed regions (fig. 1). Given that hydrophobic cores are highly conserved, most mutations likely do not significantly affect protein folding per se. However, a recent study revealed that a single peripheral mutation, (Q33E) in human Pin1 unexpectedly caused significant loss of thermostability, reinforcing that the process of detecting sensitive mutations is not a straightforward task (Zhang et al. 2018). Additionally, surface-exposed mutations potentially can affect posttranslational modification (PTM) patterns and protein function if they are located in regions that are key for interactions with other proteins and ligands.

PTMs known to modify coronavirus proteins via the addition of functional groups include glycosylation, phosphorylation, lipidation, ubiquitination, and SUMOylation (small ubiquitin-like modifier) (Fung and Liu 2018). In SARS-CoV, the four structural proteins, the auxiliary proteins, ORF3a and ORF8, and the nonstructural protein, nsp16, are known to contain PTMs. Our analyses show that most of the known sites of PTM in SARS-CoV proteins are preserved in SARS-CoV-2 proteins, whereas possible additional PTM sites still need to be explored. For example, the M glycoprotein is highly conserved between SARS-CoV and SARS-CoV-2, including the single N-glycosylation site of SARS-CoV-2 (Asn^4^) (Voss et al. 2009). However, the adjacent Ser^4^ insertion in SARS-CoV-2 is a potential site of O-glycosylation, for example. Characterization of PTMs, like glycosylation and phosphorylation, and tracking intra- and interspecies pattern variation can be of critical importance to the design of effective vaccines.

Given its crucial importance to virulence, we further examined the functional impact of substitutions between the SARS-CoV and SARS-CoV-2 S protein, which has been broadly discussed with static structures (Andersen et al. 2020; Wrapp et al. 2020; Yan et al. 2020). Here, we also use MD simulations to better understand the interactions between the S protein and host receptor(s). We also highlight the analysis of the similarities and differences of nsp1, nsp3, and nsp5 between SARS-CoV and SARS-CoV-2.

### Insights from MD Simulations about Substitutions/Conservation in the Spike Glycoprotein

The spike glycoprotein is encoded by all coronaviruses and it is necessary for the virus to enter host cells. These highly glycosylated proteins protrude from the viral surface to interact with the host cell receptor(s), which stabilizes it in a conformation (“up”) that exposes proteolytic cleavage sites. Shedding of the S1 subunit (the “cap” of S) with the action of proteases is essential to initiate the fusion of viral and host cell membranes. Given the exposure in the virion surface and its essential role for cell infection, extensive work has been performed in the structural characterization of S (Watanabe et al. 2020; Wrapp et al. 2020; Yan et al. 2020).

**Fig. 2.**
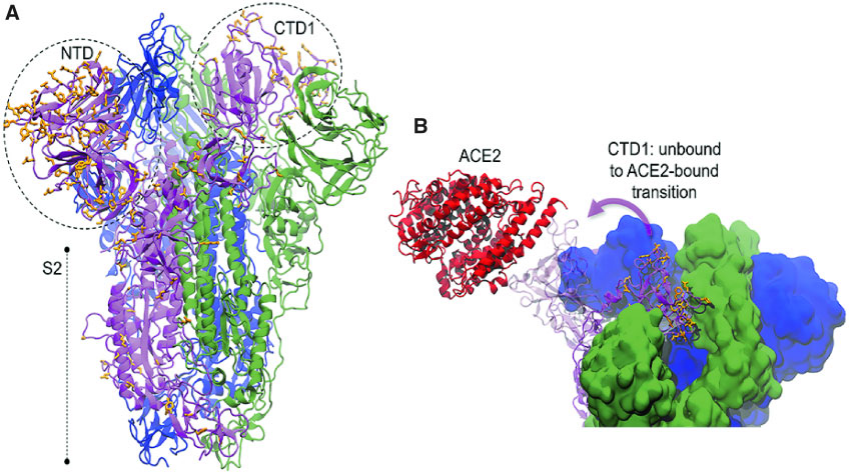
Nonconservative substitutions in the spike glycoprotein. (*A*) Local modeling-derived SARS-CoV-2 spike glycoprotein (based on PDB ids 6acc and 6ack). (*B*) Conformational transition of the receptor-binding domain of the S1 subunit of the spike glycoprotein and association with ACE2 receptor. Nonconservative substitutions relative to SARS-CoV S are depicted in *orange*. N-terminal (NTD) and C-terminal domains (CTD) are identified.

The structure of the SARS-CoV-2 S trimer has now been determined via cryo-EM, its RBD bound to ACE2 is solved at high resolution, and models of the glycosylated trimer are also available. The availability of this detailed structural information allows valuable insights regarding the functional relevance of the variation in SARS-CoV-2 S. SARS-CoV and SARS-CoV-2 S proteins are 77% identical. Most of the nonconservative substitutions are located at the N-terminal domain (NTD, fig. 2*A*), which includes the addition and deletion of N-glycosylation sites with the substitutions Asp^17^Asn and Asn^27^Ala. Other N-glycosylation sites known to be altered correspond to the mutations +Asn^74^, +Asn^149^, Asp^157^Asn, Asn^69^His, and Asn^112^Ser. The mutation Ser^323^Thr does not affect the low occurrence of O-glycosylation observed at this site (Watanabe et al. 2020). In addition to glycosylation, S is also known to undergo palmitoylation at cysteines in its cytoplasmic portion. Mutational analysis of these cysteines in SARS-CoV S revealed that palmitoylation is necessary for cell–cell fusion (McBride and Machamer 2010). The substitution Ala^1247^Cys adds a potential palmitoylation site to SARS-CoV-2 S and, thus may have an effect in S-mediated cell fusion.

Although fairly conserved, the RBD harbors a high concentration of nonconservative substitutions (supplementary table S1, Supplementary Material online, shows the conservative substitutions considered in this study), remarkably at regions that are known to directly bind to the host receptor (fig. 2*B*). These differences are expected to considerably influence the affinity of S for the host receptor, ACE2 (Hoffmann et al. 2020). Tian et al. measured the binding of the receptor-binding domain of SARS-CoV-2 S (RBD2) to ACE2 with a biolayer interferometry binding assay and reported similar affinity of RBD2 and the receptor-binding domain of SARS-CoV S (RBD1) to ACE2 (*K*_d_ = 15.0 and 15.2 nM, respectively) (Tian et al. 2020). In contrast, Wrapp et al. reported a 10- to 20-fold higher affinity of RBD2 to ACE2, compared with RBD1 (Wrapp et al. 2020). Here, we explore the effects of these substitutions in the interaction with ACE2 with MD simulations.

#### Interaction with the Host Receptor, ACE2

Applying computational methods of molecular biophysics can be a cost-effective way of identifying the key molecular elements of the virus that interact with the main receptor of SARS-CoV and SARS-CoV-2, which can be further explored with experimentation. Currently, high-resolution structures of the RBDs of the SARS-CoV and SARS-CoV-2 spike glycoproteins in complex with the peptidase domain of ACE2 are available and enable a detailed description of the interfacial interactions (Yan et al. 2020). We used these structures as a starting point for a comparative atomistic MD study of the RBDs of the two viruses in complex with ACE2, here referred as RBD1-ACE2 and RBD2-ACE2 for SARS-CoV and SARS-CoV-2, respectively.

**Fig. 3.**
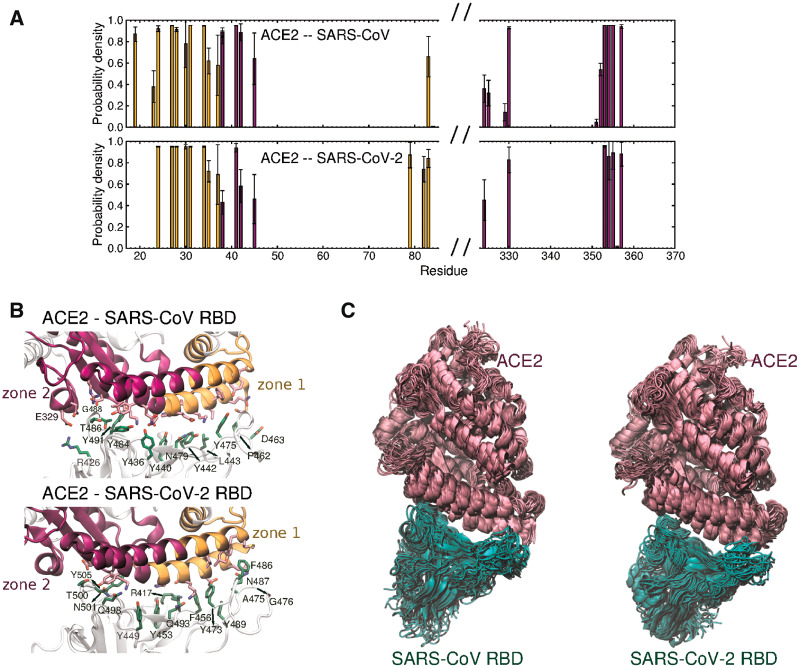
Analysis of simulations of SARS-CoV and SARS-CoV-2 RBDs in complex with ACE2. (*A*) Probability density of residues from ACE2 forming contacts with the RBDs. A maximum distance of 4 Å between any atom in a pair of residues was established. Bars with a standard deviation >50% of the probability density are considered transient contacts in the simulations and are not included in these plots. The colors of the bars correspond to zone 1 and zone 2 of ACE2, defined in (*B*), which shows the RBD residues involved in contacts formed during more than 70% of the simulation time (labeled in *green*). RBD and ACE2 residues are represented as licorices, in *green* and *pink*, respectively. (*C*) Superimposition of frames in a representative simulation of RBD1-ACE2 (*left*) and RBD2-ACE2 (*right*), using the initial position of ACE2 as reference for alignment. RBDs and ACE2 are represented in *green* and *pink*, respectively.

RBD1-ACE2 and RBD2-ACE2 complexes are stable relative to their initial configuration during all of the conducted MD simulations. The computed average number of contacts (residues with Cα <8 Å distant) is the same between RBD2 and ACE2 (23 ± 2) than in the complex with RBD1 (23 ± 2) (supplementary fig. S1, Supplementary Material online). This suggests that, if RBD2 has a higher affinity for ACE2 than RBD1, as reported by Wrapp et al., this is the result of stronger rather than additional interactions in RBD2-ACE2. As shown in figure 3*A*, the profile of ACE2 residues involved in persistent interactions with the RBDs is consistent in triplicate simulations. The contact profile, figure 3*A* and *B*, shows a slightly higher density of stable contacts in zone 2 for RBD1-ACE2 compared with RBD2-ACE2, that is likely partially due to the additional salt bridge formed by RBD1 Arg^426^ and ACE2 Glu^329^, which is lost with the substitution Arg^438^Asn in RBD2, as well as due to the presence of RBD1 Tyr^484^ (Gln^498^ in RBD2), packing with the hydrophobic tail of ACE2 Lys^353^. The weaker interactions in zone 2 are at least partially compensated in RBD2-ACE2 in zone 1, where hydrophobic packing is enhanced by the bulky RBD2 Phe^486^ and Phe^456^ (Leu^472^ and Leu^443^ in RBD1, respectively).

The analysis of the conformational dynamics of the two complexes can reveal effects of structural differences that analyses of static structures may not. From a close inspection of the structures, we find the substitution of Lys^447^ by Asn^460^ in RBD2, which results in the loss of a salt bridge with Asp^407^, or Asp^420^ in RBD2 (supplementary fig. S2, Supplementary Material online). We hypothesized that the weaker interaction with the α3 helix “unlocks” loop β4–5, that mostly interacts with zone 1 of ACE2. The elongation of the loop with the additional glycine, Gly^482^, may further contribute to the higher conformational flexibility of the SARS-CoV-2 RBD. As shown in the superimposition of frames (fig. 3*C*), the simulations suggest that this substitution does not present a significant effect in the loop mobility for the given temperature, 310 K. However, further studies at higher temperature may reveal possible consequences of these mutations to the thermostability of S and to the binding affinity to ACE2.

Neither SARS-CoV nor SARS-CoV-2 RBDs are glycosylated near the interface with the receptor, but ACE2 Asn^90^ is known to be a N-glycosylation site. In the crystallographic structure of RBD2-ACE2 complex (Protein Data Bank [PDB] id 2ajf), a trisaccharide is found attached to this site. We computed the statistics of hydrogen bonds between this glycan and amino acid residues in RBD1 and RBD2 and verified that, in both systems, the terminal glycan (β-mannose) interacts mostly with the equivalent residues Thr^402^/Thr^415^ during 9% and 14% of the simulation time, respectively (supplementary fig. S3, Supplementary Material online). Supplementary simulations of the nonglycosylated RBD2-ACE2 suggest that these interactions may have only marginal effects on complex stability. The average root mean square deviation of Ca atoms in the RBD2 relative to the crystallographic structure is 4.8 ± 1.6 and 4.7 ± 1.2 Å for glycosylated and nonglycosylated complexes, respectively.

#### ACE as a Secondary Receptor for SARS-CoV

Although considerable work has been carried out confirming that ACE2 is an efficient host receptor for SARS-CoV and SARS-CoV-2, it is unclear if other receptors can play a similar role. Transcriptome-wide gene expression data indicate that the lung, which is widely reported as the major conduit for entry of SARS viruses, expresses little to undetectable levels of ACE2 (gtexportal.org). Closer scrutiny of the initial reports identifying ACE2 as the receptor for SARS-CoV evokes the hypothesis that this result is specific to kidney-derived cell lines because ACE2 is highly expressed in this organ (supplementary text, Supplementary Material online). In contrast, a homolog to ACE2, ACE, is highly expressed in the lung and has been shown to increase infection of SARS-CoV when overexpressed in some cell types (Nie et al. 2004). A potential role of ACE as a receptor for coronaviruses therefore has not yet been fully evaluated.

As a preliminary test of the hypothesis that ACE is an alternative receptor for SARS-CoV and SARS-CoV-2, we also conducted MD simulations of RBD1-ACE and RBD2-ACE. The peptidase domains of ACE and ACE2 are 40% identical and have a very similar fold (RMSD 6.6 Å) and therefore we assumed that the interaction with RBDs would occur in the same region in the protein fold. We built the initial structures by alignment and replacement of ACE2 by ACE in the complexes described above. On the putative complex interface, only 35% of the residues are similar or identical to residues identified as stable in the interaction of ACE2 with RBD1 or RBD2. Therefore, local structural rearrangements are expected to happen in the built RBD1/2-ACE complexes during the MD simulations. In order to allow structural adjustments to happen, we conducted long equilibration simulations involving multiple steps for a gradual relaxation of the system.

**Fig. 4.**
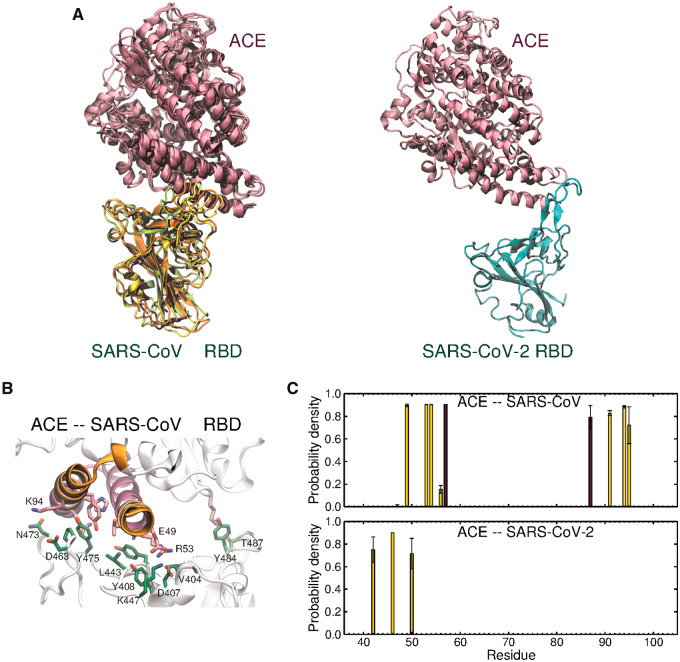
Analysis of simulations of SARS-CoV and SARS-CoV-2 RBDs in the built complex with ACE. (*A*) Superimposition of the last frames of the simulations of RBD1-ACE (*left*). For visual clarity, because the relative orientation of the proteins in RBD2-ACE is very flexible due to the small surface of contact, we only show the last frame of a representative simulation of RBD-ACE (*right*). RBDs and ACE are represented in *green* and *pink*, respectively. (*B*) Residues involved in contacts formed during more than 70% of the simulation time. RBD and ACE residues are represented as licorices, in *green* and *pink*, respectively. (*C*) Probability density of residues in ACE forming contacts with the RBDs. A maximum distance of 4 Å between any atom in a pair of residues was established. Bars with a standard deviation higher than 50% of the probability density are considered transient contacts in the simulations and not included in these plots. The colors of the bars correspond to zone 1 and zone 2 of ACE, shown in (*B*).

In both systems, the RBDs remain bound to ACE during the simulations. In all independent simulations of RBD1-ACE, a significant reorientation of the RBD1 is observed, so that the loop β5–6 slides toward the center of the α1 helix of ACE. Figure 4*A* shows the superimposed last frames of the three simulations of RBD1-ACE. Persistent interactions are established involving the formation of three salt bridges, namely, Asp^407^–Arg^53^, Lys^447^–Glu^49^, Asp^493^–Lys^94^, from RBD1 and ACE, respectively (fig. 4*B*).

The RBD2-ACE also converges to a common configuration in two of the three independent simulations of RBD2-ACE, with only a few residues attaching the proteins together (fig. 4*A*). In these simulations, the loop β4–5 anchors the RBD2 to the N-terminal of α1 helix and the nearby region of α2 helix of ACE, mostly involving only hydrophobic contacts between Phe^456^ and Tyr^489^ of RBD2 at the N-terminal of α1 (fig. 4*C*).

Despite the fact that MD simulations of hundreds of nanoseconds cannot provide reliable quantitative estimates of binding affinity, they can be effectively used as a preliminary method to explore the relative stability of the studied complexes. Taken together, our simulations demonstrate the convergence of stable and strong interactions between ACE and SARS-CoV, suggesting that ACE may allow for infection in tissues with low or undetectable levels of ACE2 and high ACE expression. Notably, this is in line with in vivo results that suggest that SARS-CoV can replicate slightly better in human lungs than SARS-CoV-2 in early stages of infection (Hui et al. 2020). In contrast, the simulations do not provide strong support for the hypothesis of ACE acting as a receptor for SARS-CoV-2. No variants of ACE with potentially increased binding affinity to RBD2 (i.e., variants with higher similarity to ACE2 in the interfacial region) were identified in the nonsynonymous SNPs listed in dbSNP at NCBI. However, we emphasize that this hypothesis has to be thoroughly evaluated through experiments designed to include the complete native spike protein since intraspike interactions of the RBD in the closed conformation are an important element that competes with the stabilization of the open conformation of the spike via interaction with the host receptor.

### Functionally Relevant Substitutions/Conservation in nsp1, nsp3, and nsp5 Proteins

We also highlight molecular differences and similarities of nsp1, nsp3, and nsp5 proteins between SARS-CoV and SARS-CoV-2 as they relate to host immune response and to pathogenicity divergence, being promising targets for drug development, drug repurposing, or vaccine production.

#### Nonstructural Protein 1 (nsp1)

Nsp1 is the first nonstructural protein coded in the ORF1a/ORF1ab gene. In vitro experiments suggest that SARS-CoV nsp1 disrupts the host interferon defense response by potentially affecting downstream signaling (Züst et al. 2007; Narayanan et al. 2008, 2015). It also binds the 40S ribosomal subunit, which has been associated with degradation of host mRNA and suppression of host mRNA translation, leaving the viral RNA unaffected (Züst et al. 2007; Kamitani et al. 2009). The resultant complex cleaves the 5′ UTR of host mRNAs, inhibiting translation.

**Fig 5.**
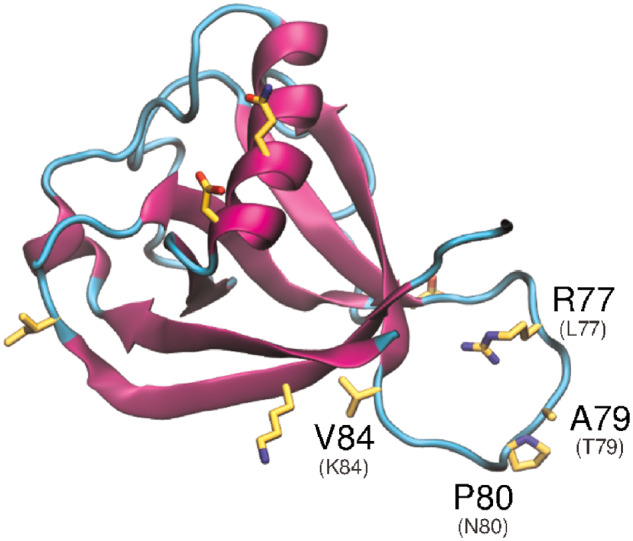
Analysis of the structural variation in SARS-CoV-2 nsp1 protein relative to SARS-CoV nsp1. Fragment-based predicted structure of nsp1. Nonconservative substitutions relative to SARS-CoV nsp1 are depicted in *yellow*. Substitutions discussed in the text are labeled, including the corresponding residue of the homolog (SARS-CoV) in parentheses.

Nsp1 is highly conserved between SARS-CoV and SARS-CoV-2. Notably, in SARS-CoV-2, there are four substitutions in the less conserved β3–4 loop (fig. 5), namely, Leu^77^Arg, Thr^79^Ala, Asn^80^Pro, and Lys^84^Val. These substitutions may directly relate to pathogenicity as experimentally induced substitutions in the same region (Arg^73^Glu, Asp^75^Arg, Leu^77^Ala, Ser^78^Glu, and Asn^80^Gly) in SARS-CoV demonstrated increased inhibition of host gene expression and antiviral signaling, compared with the SARS-CoV wild type (Jauregui et al. 2013). Subsequent experiments in mice showed that the deletion of this loop in SARS-CoV resulted in an increased survival rate and less severe lung damage (Jimenez-Guardeño et al. 2015). Given that this loop plays an essential role in the ability of nsp1 to impair host-translational activity, and the three substitutions in SARS-CoV-2 may be important elements of virulence divergence, this should be targeted in future studies that focus on disrupting infection.

#### Nonstructural Protein 3 (nsp3)

**Fig. 6.**
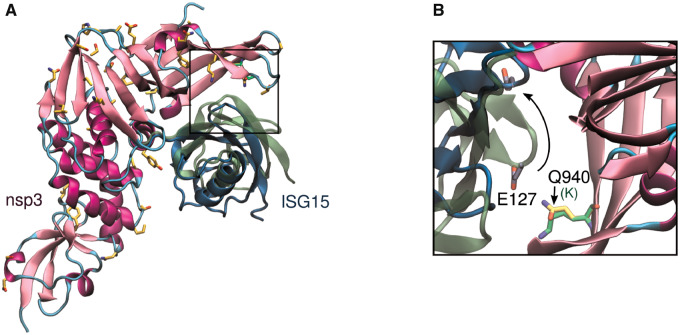
Papain-like protease domain of SARS-CoV-2 nsp3 bound to human ISG15. (*A*) Nonconservative substitutions relative to SARS-CoV are depicted in *orange* in PL2^Pro^ (structure in *pink*). ISG15 bound to SARS-CoV-2 PL2pro (PDB id 6xa9) is represented in *blue*, and ISG15 bound to SARS-CoV PL2^Pro^ (PDB id 5tl6) is represented in *green*. (*B*) The substitution of Lys^917^ (in *green*) in SARS-CoV PL2^Pro^ by Gln^940^ in SARS-CoV-2 may result in the loss of a salt bridge with Glu^127^ in ISG15. The distance between side chain atoms, N_Lys^917^-O_Glu^127^ and N_Gln^940^-O_Glu^127^, is 7 Å and 13 Å, respectively.

Nsp3 is a multidomain and multifunctional protein of coronaviruses. Particularly, the papain-like protease domain (PL2^pro^) displays a key role in cleaving the viral polyprotein and suppressing the host immune response by inhibiting components that interact with the nuclear factor transcription factor-kappa B (NF-κB), interferon-beta, and p53. In a structural study, PL2^pro^ was found to bind ubiquitin-like interferon-stimulated gene product 15 (ISG15) (Daczkowski et al. 2017), the latter an important posttranslational modifier of host antiviral proteins, including cytokines like interferon. It is believed that cleaving these PTMs of cytokine proteins by PL2^pro^ disrupts the host immune response (Daczkowski et al. 2017). Importantly, ISG15 has significant interspecies variability, potentially contributing to its very different virulence patterns among host species. The region that binds to ISG15 is mostly conserved within SARS-CoV and SARS-CoV-2, including residues that were identified as critical for the interaction with ISG15, namely Arg^911^, Met^953^, and Pro^992^ (fig. 6). However, the substitution Lys^940^Gln likely weakens the interaction with ISG15 by possibly removing a salt bridge with Glu^127^, suggesting an important mechanism for variable virulence. This hypothesis arises from the analysis of the recently solved crystallographic structure of PL2^pro^ in complex with the C-terminal domain of ISG15, shown in figure 6, and its structural alignment with the corresponding complex of SARS-CoV PL2^pro^. The reorientation of ISG15 relative to PL2^Pro^ and the increased distance between Gln^940^ in PL2^Pro^ and Glu^127^ in ISG15 are remarkable. Experiments or extensive MD simulations can be done to quantify the binding affinity of these proteins in the presence of Gln^940^.

#### Nonstructural Protein 5 (nsp5 or 3CLpro)

The nonstructural protein 5 (nsp5, also known as 3CL^pro^) is the main protease of the coronavirus genome that cleaves the polyproteins translated from the viral RNA into functional units (Ziebuhr et al. 2000; Anand et al. 2003; Perlman and Netland 2009). This protein is highly conserved relative to SARS-CoV (96% identity) and among RNA+ viruses (Nidovirales) in general, making it an attractive target for pan-antiviral drugs (Nukoolkarn et al. 2008; Dayer et al. 2017; Zhang, Lin, Kusov, et al. 2020).

**Fig. 7.**
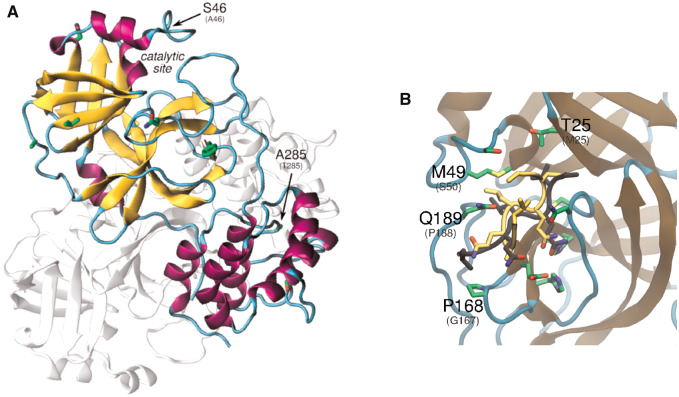
Analysis of the structural variation in SARS-CoV-2 nsp5 protein relative to SARS-CoV nsp5. (*A*) Nsp5 dimer (PDB id: 6lu7). (*B*) Close view of the catalytic site of nsp5. In *yellow*, NEMO is shown in the conformation predicted with docking. The conformation of NEMO transferred from PEDV 3CL^pro^ is also depicted, in *black* (PDB id: 5zqg). Substitutions discussed in the text are labeled, including the corresponding residue of the homolog (PEDV) in parentheses.

Studies with SARS-CoV show that dimerization is essential to stabilize the productive conformation of the 3CLpro catalytic site. The recently solved structure of 3CLpro of SARS-CoV-2 (PDB id: 6y2e) confirms the dimer as its biological state (fig. 7*A*). The dimer interface is highly conserved between SARS-CoV and SARS-CoV-2, except for the nonconservative substitution Thr^285^Ala. Based on previous studies with SARS-CoV 3CL^pro^, this substitution was thought to enhance the catalytic efficiency of nsp5 by improving hydrophobic packing within monomers. However, a recent study reported only a slightly improved catalytic efficiency of SARS-CoV-2 3CL^pro^ compared with SARS-CoV 3CL^pro^ (Zhang, Lin, Sun, et al. 2020). The analysis of the phylogenetic tree derived from the aligned sequences of coronavirus from all available species reveals that alanine at site 285 defines the SARS-CoV-2 clade and three bat coronaviruses from mainland China (supplementary fig. S3, Supplementary Material online). In contrast, many of the beta coronaviruses that infect mammals have a cysteine at this location. Given the proximity with the cysteine in the opposite monomer, it is possible that a disulfide bridge is formed in these proteases, which may result in a more tightly bound dimer and increased catalytic efficiency. Further exploration of this site is warranted.

In addition to its role in processing the viral proteome, we propose that the highly conserved nsp5 protein may also be part of a major mechanism that suppresses the NF-κB pathway, eliminating the host cell’s interferon-based antiviral response. In SARS-CoV, several proteins have been reported to be interferon antagonists, including nsp1 and nsp3 (Wathelet et al. 2007; Frieman et al. 2009). An additional mechanism of circumventing the interferon antiviral response is described for the porcine epidemic diarrhea virus (PEDV) as well as noncoronaviruses (Huang et al. 2014), in which the 3CL^pro^ cleaves the NF-κB essential modulator (NEMO) (Wang, Fang, et al. 2016). Given that the substrate-binding site of SARS-CoV-2 3CL^pro^ is very similar to PEDV 3CL^pro^, it is possible that SARS-CoV-2 3CL^pro^ is also active toward NEMO. Structural divergence is concentrated in the region corresponding to the S2 binding site of PEDV and in the peptide segment 45-51, in the catalytic entrance (fig. 7*B*). As a preliminary test for this hypothesis, we conducted molecular docking of NEMO targeting SARS-CoV-2 and PEDV 3CL^pro^ proteins. The best-ranked resulting substrate conformation has the Gln^231^ reaction center of NEMO positioned very similarly to the PEDV 3CL^pro^-NEMO crystal structure. The estimated binding affinity is −6.2 kcal/mol for SARS-CoV-2 3CL^pro^-NEMO and −7.4 kcal/mol for PEDV 3CL^pro^-NEMO. The binding site of SARS-CoV-2 3CL^pro^ is conserved relative to SARS-CoV 3CL^pro^, except by the substitution Ala^46^Ser in the entrance of the cleft, indicating that SARS-CoV 3CL^pro^ may also be active toward NEMO. This result suggests that drug development targeting this mechanism may prove fruitful as it would allow for a normal host immune response to combat the pathogen and given the conserved nature of the protein across diverse coronaviruses efficient inhibitors may potentially be broad-acting.

## Concluding Remarks

A global understanding of the genetic determinants of viral pathogenesis can be built from a Systems Biology approach integrating virus-centric, host-centric, and virus–host interaction layers of information. However, the effectiveness of mapping multiomics interactions greatly depends on a consolidated set of knowledge about the role of host and virus genes and their products. For example, studies focused on the NS1, hemagglutinin, and neuraminidase proteins reveal that they are key components determining virulence of the influenza virus (Kash et al. 2004; Hale et al. 2008). These results served as the basis for systems-level studies exploring correlations between recombinant viruses and host responses (Korth et al. 2013).

Similarly, genomic approaches have been performed attempting to elucidate the evolutionary origins of SARS-CoV-2. Using integrated comparative genomics and machine learning techniques, Gussow et al. identified an enhancement of nuclear localization signals in the nucleocapsid protein and inserts in the spike glycoprotein as potential dominant genomic features that contribute to the higher case fatality rate of SARS-CoV, SARS-CoV-2, and Middle East respiratory syndrome coronavirus (MERS-CoV) compared with endemic coronaviruses (Gussow et al. 2020). Given the prominent difference in transmission rate between SARS-CoV and SARS-CoV-2, such analysis is augmented in the present study to suggest the molecular features that are likely major determinants of the pathogenicity differences between them.

Except for ORF8 and ORF10, which code for proteins whose functions are not currently known, the remaining proteins are highly conserved within SARS-CoV and SARS-CoV-2, sharing identity of >70%. However, this study indicates that substitutions in key functional regions of different proteins are likely modifying the interaction with host or viral proteins, and these local effects may be responsible for the distinct pathogenic profile of SARS-CoV-2. In contrast, we identified highly conserved and functionally important regions in proteins, such as the main protease (3CLPro), that are promising targets for the development of broad-spectrum antivirals. In order to test the hypotheses raised in this study, a set of future experiments should be designed to determine the exact impact of molecular conservation/differences in SARS-CoV-2 virulence. For example, in vitro experiments could be performed to test the proposed interaction of 3CLPro and NEMO and the binding affinity difference between SARS-CoV and SARS-CoV-2 PL2Pro with ISG15. Further study is also required to verify the hypothesis of ACE being a secondary receptor for SARS-CoV in the absence of ACE2 and the possible effects in the incidence of respiratory distress in SARS compared with COVID-19. Finally, similar to methods using recombinant viruses to study determinants of r1918 virulence (Geiss et al. 2002; Kash et al. 2004; Billharz et al. 2009), mutants of SARS-CoV and SARS-CoV-2 can be generated from the genes highlighted here to assess the magnitude of their contribution to virulence.

Although not a traditional protocol, advances in computational power and methods can make comparative proteome-wide structural analysis a valuable approach to research on viral pathogenesis. Additionally, the same workflow developed for this study could help to identify specific mutations that allowed for the bat-to-human leap of SARS-CoV-2, which is of great importance to prepare strategies against future zoonosis from novel coronaviruses. A strong hypothesis for the origin of SARS-CoV-2 seems to be that a closely related bat virus, such as RaTG13, was able to infect humans, and natural selection, possibly driven by cytosine deamination, favored the high human-to-human transmission of SARS-CoV-2. In fact, nearly half of nonsynonymous mutations between RaTG13 and SARS-CoV-2 comprises C > U transitions (Matyášek and Kovařík 2020). Comparative protein structural analysis in future studies could help to elucidate their contribution to SARS-CoV-2 virulence among humans.

As part of this effort, we provide the extensive structural analysis of the viral proteome, all of which is available as a web resource (https://compsysbio.ornl.gov/covid-19/covid-19-structome/, last accessed September 16, 2020) and in supplementary figures S5–S24, Supplementary Material online. We also make available, in the webpage, the predicted models and lists of SARS-CoV versus SARS-CoV-2 substitutions that can be easily projected in the protein structures for further analysis. The collective analysis also informs the identification of promising drug, vaccine, and diagnostic targets for COVID-19.

## Materials and Methods

### Ensemble Workflow for Protein Structure Prediction

To date, partial or full structures of five proteins from SARS-CoV-2 have been experimentally solved. In view of the urgency to understand the molecular machinery of SARS-CoV-2, we used an ensemble workflow to generate structural models of all unsolved structural and mature nonstructural viral proteins. Due to the performance of methods for protein structure prediction varying by complexity, protein sequences were carefully analyzed to optimize the combination of the state-of-the-art methods of protein structure prediction. As such, the resulting models have the highest possible resolution and maximum information regarding the overall shape of each protein. Here, we provide a synopsis for each of the 27 mature viral proteins including structural models, variability relative to SARS-CoV, and the potential functional relevance to SARS-Cov-2.

Case-by-case protocols were generated based on a profile extracted from each sequence, consisting of two main factors:

1. *Primary sequence-based information.* Residues within conserved domains (Pfam [Finn et al. 2014]) and intrinsically disordered regions were identified using IuPred2 (Mészáros et al. 2018), which relies on the composition of amino acid segments and their tendency to form stable structural motifs. TMHMM (Krogh et al. 2001) was used to predict the helical transmembrane protein regions based on a hidden Markov model. No β-barrel transmembrane proteins are coded for in SARS-CoV-2.
2. *Availability of experimentally determined structures.* PSI-BLAST was used to identify homologous with partial or full structures available in the PDB that could be used as templates for modeling.

Several SARS-CoV proteins that are highly conserved have been solved experimentally and were available for our analysis. In order to maximize the accuracy of translating information from these structures, amino acid substitutions were analyzed to identify those that likely impact protein conformation. Examples of changes that affect protein structure are a hydrophobic side chain being replaced by a charged amino acid at the protein core or a substitution to proline (a helix “breaker”) within a helical structure. In case such substitutions are not found, and the protein has more than 70% identity to the template, loops and substitutions are locally modeled (LM) using the Rosetta remodel (Huang et al. 2011) and fixbb (Kuhlman and Baker 2000; Hu et al. 2007) applications, respectively. The comparison of recently released crystallographic structures with the models generated using carefully analyzed protein sequences and LM for selected regions appears to be an effective approach (supplementary table S3, Supplementary Material online). Achieving high local resolution, especially in sites of substrate/ligand binding, can considerably enhance the results of subsequent studies for small molecule candidate identification using molecular docking. Although ensemble docking approaches are often applied to contend with the conformational flexibility of the protein target, refining the binding site based on structural information from homologs in the *holo* form, if available, is more suitable for identifying functional complexes.

Homology-based modeling is typically the optimal approach for cases in which the identity to the template is above 30%. The fragment-based approach of the I-TASSER (Yang et al. 2015) workflow was used in cases where the range of identity was 30–70%, and to provide an alternative model to LM in regions of proteins harboring substitutions that would be expected to significantly affect protein conformation. In order to predict structures for proteins that do not have a crystal structure of a homolog available, we applied the trRosetta (Yang et al. 2020) workflow. Based on benchmarks of the Critical Assessment of Techniques for Protein Structure Prediction (CASP13), trRosetta was designed to achieve sound performance for modeling novel folds by using a deep residual network for predicting interresidue distance and orientation that guides energy minimization. We use the analysis of nsp3, the largest mature protein of SARS-CoV-2, as an example of the workflow (supplementary fig. S4, Supplementary Material online).

### MD Simulations

The solved structures of the complexes of SARS-CoV and SARS-CoV-2 RBDs bound to ACE2, referred here as RBD1-ACE2 and RBD2-ACE2, respectively, were used as starting configuration for atomistic MD simulations (PDB ids: 6m17 and 2ajf) (Li et al. 2005; Yan et al. 2020). MD simulations were performed with the 2020 version of GROMACS (Lindahl and Hess 2020). Five and three independent simulations were performed for each complex with ACE2 and ACE, with a total of 500 and 300 ns of production per system, respectively. The CHARMM36 protein force field (Huang and MacKerell 2013) was used with TIP3P water (Jorgensen et al. 1983). The CHARMM carbohydrate force field was applied for glycans (Guvench et al. 2011). CHARMM-GUI was used to prepare the simulation inputs (Jo et al. 2008). The full simulation protocol is described in the Supplementary Material online.

### Molecular Docking

Molecular docking of the NEMO targeting SARS-CoV-2 and PEDV 3CL^pro^ proteins was performed using Autodock Vina (Trott and Olson 2010). Autodocktools were used to prepare the inputs (Morris et al. 2009). The search space was defined as a box with dimensions 20 × 20 × 20 Å, encompassing the side chains of the full catalytic site of these enzymes. Grid space 1.0 Å was used, and exhaustiveness parameter was set 20. The N-Cα and Cα-C bonds in the segment Gln^229^–Ala^233^ and bonds in the side chains of Leu^227^, Leu^230^, Val^232^, and Ala^233^ were set as flexible, except for those forming π-conjugated systems. The remaining bonds were fixed in the conformation of NEMO in the crystal structure of PEDV nsp5-NEMO (PDB id 5zqg), as the results of Vina are often more accurate for a number of active bonds lower than 15 (Wang, Sun, et al. 2016).

## Supplementary Material

Supplementary data are available at *Molecular Biology and Evolution* online.

## Supplementary Material

msaa231_Supplementary_Data

## Notes

This manuscript has been authored by UT-Battelle, LLC under contract no. DE-AC05-00OR22725 with the U.S. Department of Energy. The United States Government retains and the publisher, by accepting the article for publication, acknowledges that the United States Government retains a nonexclusive, paid-up, irrevocable, world-wide license to publish or reproduce the published form of this manuscript, or allow others to do so, for United States Government purposes. The Department of Energy will provide public access to these results of federally sponsored research in accordance with the DOE Public Access Plan (http://energy.gov/downloads/doe-public-access-plan, last accessed September 16, 2020).
