# Supplementary material for: Potential Pathogenicity Determinants Identified from Structural Proteomics of SARS-CoV and SARS-CoV-2
Source: Mol Biol Evol. 2020 Sep 17;38(2):702–15. doi: 10.1093/molbev/msaa231 (PMC7543629; doi:10.1093/molbev/msaa231)
Supplement: msaa231_Supplementary_Data [file msaa231_supplementary_data.pdf]

Supplementary Material

Potential pathogenicity determinants identified from structural proteomics of SARS-CoV and SARS-CoV-2

Authors: Erica T. Prates[1,2‡], Michael R. Garvin[1,2‡], Mirko Pavicic[1,2], Piet Jones[2,3], Manesh Shah[6], Omar Demerdash[1], B Kirtley Amos[4], Armin Geiger[2,3], Daniel Jacobson[1,2,3,5,6*]

[‡]**These authors contributed equally to this work.**

[*]**Corresponding author. E-mail: jacobsonda@ornl.gov**

Affiliations: [1]Oak Ridge National Laboratory, Biosciences Division, Oak Ridge, TN; [2]National Virtual Biotechnology Laboratory, US Department of Energy; [3]The University of Tennessee Knoxville, The Bredesen Center for Interdisciplinary Research and Graduate Education, Knoxville, TN. [4]University of Kentucky, Department of Horticulture, N-318 Ag Sciences Center, Lexington, KY. [5]The University of Tennessee Knoxville, Knoxville, Department of Psychology, TN. [6]The University of Tennessee Knoxville, Knoxville, Genome Science and Technology, TN.

1. Supplementary Figures

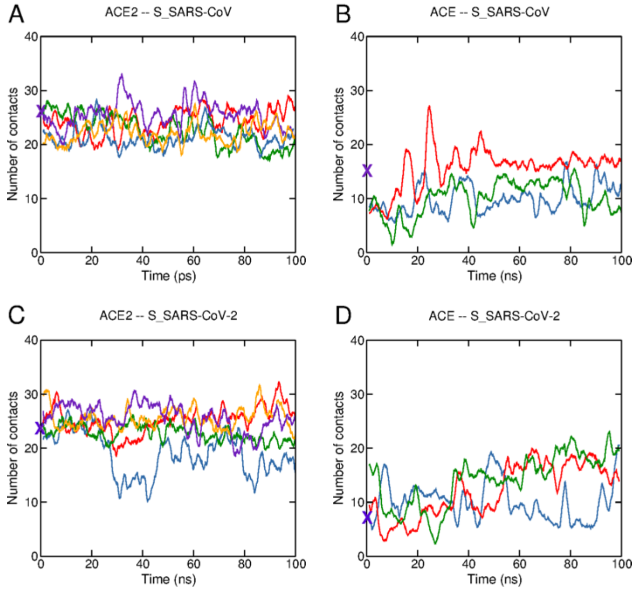

Fig. S1.

Time evolution of the number of contacts within the receptor-binding domain (SARS-CoV and SARS-CoV-2 spike glycoproteins) and the putative receptors ACE2 and ACE. A contact was considered for pairs of residues with C-alpha less than 8 Å distant. The colors of lines indicate the different independent simulations (three or five for each system) and the *purple* "X" indicates the number of contacts in the initial conformation.

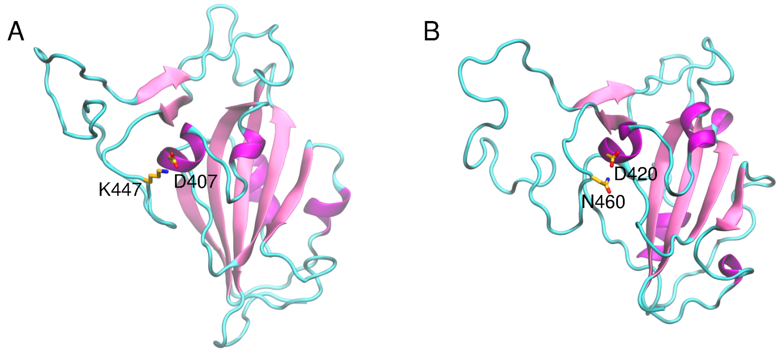

Fig. S2.

Substitution of Lys^{447} in SARS-CoV S protein (A) by Asn^{460} in SARS-CoV-2 S (B) protein results in the loss of a salt bridge connecting a long loop to RBD core.

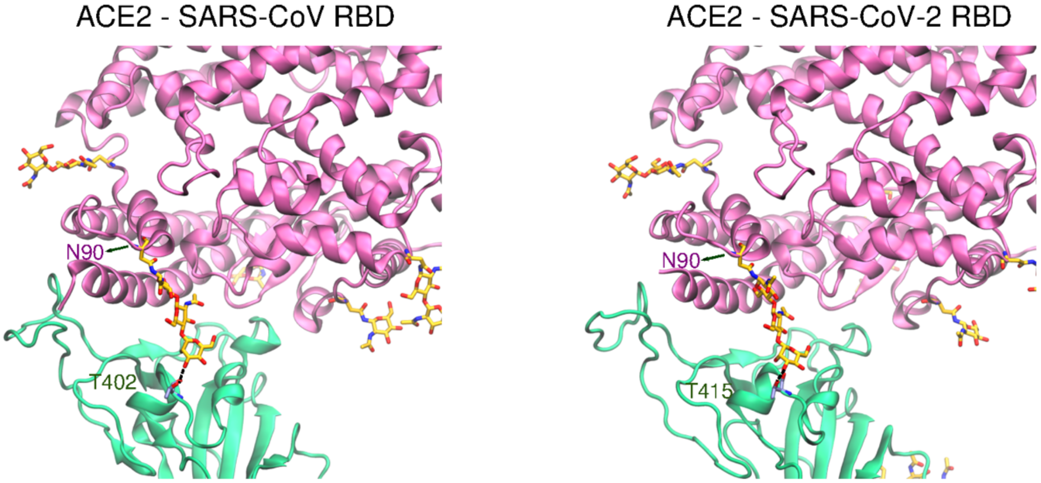

Fig. S3.

Interaction of glycan attached to Asn^{90} with Thr^{402} and Thr^{415} in RBD1 and RBD2.

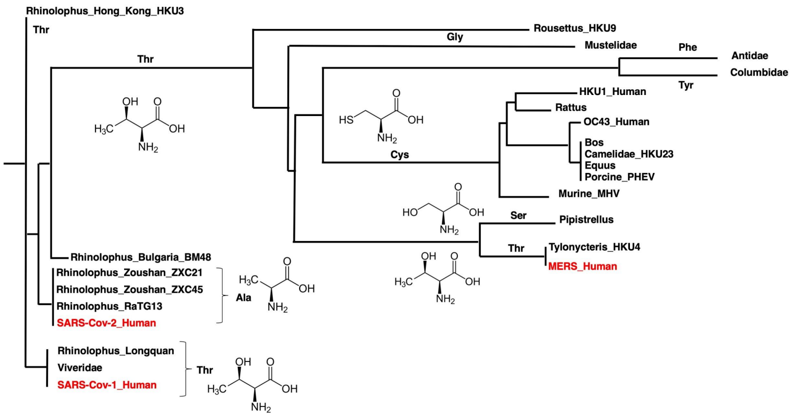

Fig. S3.

Phylogenetic tree of all known beta coronaviruses using the full protein sequence for 3CL^{pro}. *Red* text indicates viruses of concern for human health. The amino acid changes for the site at 285, which may affect dimerization are shown along branches. An alanine at this site defines the SARS-CoV-2 clade with the horseshoe bats from mainland China.

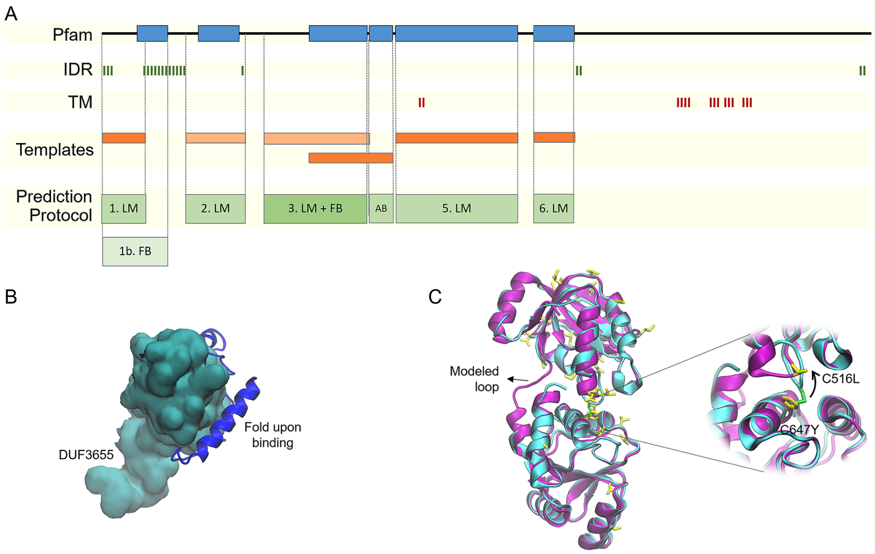

Fig. S4.

Ensemble workflow for structure prediction of SARS-CoV-2 nsp3. Case-by-case protocols of structure prediction are determined by finely parsing each protein sequence using information about the position of intrinsically disordered regions (IDR), transmembrane regions (TM), signal peptides, and templates (A). The method applied to SARS-CoV-2 nsp3 defined six regions to be modeled by the local modeling (LM), fragment-based (FB), and/or *ab initio* (AB) approaches. The first region, 2-169, of the nsp3 sequence was modeled using the combination LM+FB (B) so that the structured region (5-111) could be determined in high resolution via LM of the side chain using a highly similar template (PDB 2gri, identity 76%). The bound conformation of intrinsically disordered segments was predicted using FB, a more flexible method. The choice of prediction method for protein regions with templates of high identity includes a thorough structural analysis of the template. For example, the region 413-676 of nsp3 is aligned with a high identity template (2w2g, 76%), so that modeling only variant side chains was considered. However, due to the predicted loss of a disulfide bridge, the FB approach was used since it allows larger conformational changes (C).

2. Supplementary Tables

Table S1.

Classification of conservative substitutions considered in this study. Amino acids in brackets can be considered to be part of a group after structural analysis.

Group	Amino acid
Aromatic	Y, W, [H], [F]
Non-polar	A, I, V, L, M, [F], [P]
Polar, hydrogen bond interacting	S, T, [C]
Amidic, hydrogen bond interacting	N, Q
Acidic	D, E
Basic	R, H, K
Glycine	G

Table S2.

Hydrogen bond interactions in the simulations (I-V) of ACE2-RBD2, in percentage (%) of time steps. The total occurrence is shown in the last column. Only interactions persisting for more than 10% of the simulation time are shown.

ACE2	RBD2	I	II	III	IV	V	TOTAL
Asp^{30}	Lys^{417}	56	32	51	55	54	49
Glu^{35}	Gln^{493}	6	10	8	13	12	10
Glu^{37}	Thr^{505}	5	10	41	21	39	23
Lys^{353}	Gly^{502}	41	11	48	50	46	39
Asp^{355}	Thr^{500}	35	9	46	32	31	30

Table S3. Average root-mean-square deviation (RMSD) of models built with the workflow relative to experimentally determined structures (*Workflow*). In parenthesis, the region modeled is defined. *Exp.*: Resolution of experimentally determined structure; *Other*: average RMSD of models generated with C-I-TASSER pipeline (Zheng et al. 2019). Structure alignment was conducted using Lovoalign (Martínez et al. 2007).

Protein	Exp. / Å	Workflow / Å	Other / Å
nsp5 (full)	2.0	1.5	2.8
nsp3 (205-374)	1.5	1.5	-
nsp3 (746-1062)	2.7	1.5	1.2
nsp15 (27-332)	2.2	0.7	0.5

3. Supplementary Text: ACE2 is not highly expressed in uninfected lung tissue

Both the Genotype-Tissue Expression (GTEx) and Proteomics DataBase (Proteomics DB) indicate ACE2 is either expressed at very low levels or is not detectable in lung, currently thought to be major entry site for SARS-CoV and SARS-CoV-2. Reports of expression of ACE2 in lung are based on mRNA or protein quantification (Harmer et al. 2002; Hamming et al. 2004; Sims et al. 2005; Tseng et al. 2005; Ren et al. 2006). The two earliest reports that measured ACE2 mRNA in lung tissue showed either no expression at all or low levels (Donoghue et al. 2000; Harmer et al. 2002), whereas ACE is found at moderate to high levels; neither were quantitative and both were based on small sample sizes.

Other early reports of high expression of ACE2 in the lung were based on protein expression using a peptide-derived polyclonal antibody and immunohistochemistry (Hamming et al. 2004; Ren et al. 2006). Aside from being non-quantitative, the results are difficult to interpret and could be due to the non-specificity of these types of antibodies. The three remaining reports of ACE2 expression in the lung or lung-derived epithelium were based on improved antibody immunohistochemistry and western blots (Jia et al. 2005; Sims et al. 2005; Tseng et al. 2005). Although still not quantitative, the reports are again consistent with GTEx and Proteome DB; ACE2 is either not expressed or found at very low levels in normal lung tissue or normal lung-derived epithelial cells. However, it is induced either by exposing cells grown in culture to air (air-liquid interface) or by infection with the SARS-CoV virus, suggesting that compromised lung epithelium or cells that are already infected are routes of infection in this tissue.

4. Supplementary Text: ACE may mediate SARS-CoV infection

A potential role of ACE as receptor for SARS-CoV is not yet fully explored. In the experiments used to identify ACE2 as the SARS-CoV receptor, ACE was not overexpressed as it was done with ACE2 (Li et al. 2003). A subsequent study using pseudotyped virus demonstrated a correlation between ACE2 expression and infectivity in several cell lines and although association with ACE2 was much higher, overexpression of ACE was shown to increase infectivity in some cell lines (Nie et al. 2004). Notably, ACE2-mediated infection was highest in kidney- and colon-derived cell lines and much less efficient in those from the lung. This same pattern in kidney-, colon-, and lung-derived cell lines was confirmed in a more recent analysis of beta-coronaviruses (Letko et al. 2020). We note that the several studies that focused on SARS-CoV infection mediated by ACE2 were conducted using kidney- and colon-derived cell lines, which express higher levels of ACE2 than ACE. In the lung and other respiratory tissues, the reverse is true; ACE is expressed at higher levels than ACE2. Experiments *in vitro* show that ACE does not bind to SARS-CoV (and perhaps SARS-CoV-2) S protein as efficiently as ACE2, but, given an environment in which levels of ACE are an order of magnitude higher than ACE2, as is the case in the lung, the potential alternative association with ACE has to be further verified.

5. Supplementary Text: Molecular dynamics simulations

Proteins were solvated with a 15 Å water layer in an octahedral box under periodic boundary conditions. Sodium and chloride ions were added at a concentration of ~0.16 M to ensure the

electroneutrality of the system. All ions present in the original crystallographic structures were kept for the simulations. The system was first energy minimized for 5000 steps with steepest descent. This was followed by two phases of equilibration. In the first phase, 6 ns passed while applying positional constraints on α-carbons except for those at binding interfaces. The temperature was gradually raised to 298.15 K. In the second phase, 20 ns passed during which positional constraints were applied to alpha carbons of the C-terminal domains of ACE and ACE2 and a few residues in the core of the spike protein's receptor-binding domain. In each simulation of the triplicate, atomic velocities were reinitialized from a Maxwell-Boltzmann distribution at 10 ns, using a different random number of seed in each case. Equilibration and production were run in the NPT ensemble, and a 2 fs time step was used. Temperature was maintained using velocity rescaling via a stochastic term that properly generates constant pressure-constant temperature ensembles (Bussi et al. 2007); and a coupling constant of 1.0 ps. The Berendsen barostat (Berendsen et al. 1984) was used with a coupling constant of 1.0 ps to maintain a pressure of 1 atm. Systems were run with a total of 300-500 ns of production.

In the analysis of the simulations, the time evolution of the number of contacts was computed using the mindist utility of GROMACS and a distance cutoff within alpha carbons of 8 Å. The probability density of contacts was computed using the timeline plugin of VMD (Humphrey et al. 1996). Visual analysis and the design of figures were made using version 1.93 of VMD.

6. Supplementary Text: Structural Analysis of the SARS-CoV-2 Proteome

Nonstructural protein 1, nonstructural protein 5 and spike glycoprotein are discussed in the main text.

Nonstructural protein 2 (nsp2)

Positive RNA viruses create viroplasms, subcellular compartments that are thought to protect the viral machinery against host-defense strategies, and even facilitate replication (Netherton and Wileman 2011). They often contain double-stranded RNA, as well as the replication complex of the virus. For SARS-CoV, the replicase proteins assemble at the cytosol side of the ER, after which invagination occurs. There is no evidence of a pore maintaining the connection of the invaginated space to the cytosol, thus no maintained sphererule has been observed, though vesicles do form. A network of double-membrane-vesicles (DMVs) is eventually formed, anchored to the ER by a convoluted-membrane (CM) compartment. Replication appears to happen in the CM, while DMVs directly connected to the CM contain replicase proteins and viral proteins. Those DMVs not directly connected to CM form vesicle packets containing viral genomes (Knoops et al. 2008; Netherton and Wileman 2011).

In Hagemeijer *et al.* (2010), it was shown that the nonstructural protein 2 (nsp2) localized to the membranes of CM and DMV, and it is exposed to the cytoplasm. It appears that during the formation of the replication complex, nsp2 is recruited, but once formed no further exchange of nsp2 occurs. Mutants of SARS-CoV with the deleted nsp2 coding sequence are still capable of viral replication, although with decreased growth (Graham et al. 2005). The specific role of nsp2 has not been established; It has been shown that nsp2 interacts directly with prohibitin 1 (PHB1)

and PHB2 (Cornillez-Ty et al. 2009) host proteins with a wide variety of functions, such as in the regulation of the mitochondrial respiratory function, autophagy, mitochondrial stability, and inflammatory response in lung and gut (Kathiria et al. (2012)Hernando-Rodríguez and Artal-Sanz (2018), (Agrawal et al. 2012; Hernando-Rodríguez and Artal-Sanz 2018)(2012). Additionally, Von Brunn *et al*. (2007) have shown via co-immunoprecipitation experiments that nsp2 interacts with several other viral proteins, such as nsp3, nsp6, nsp8, nsp11, nsp16 and ORF3a. Notably the latter is known to disrupt mitochondrial stability in SARS-CoV-1 infection (Padhan et al. 2008). These results suggest that nsp2 may be involved in mitochondrial dysfunction through PHB1 and 2, and association with LC3 may be worth investigating.

A

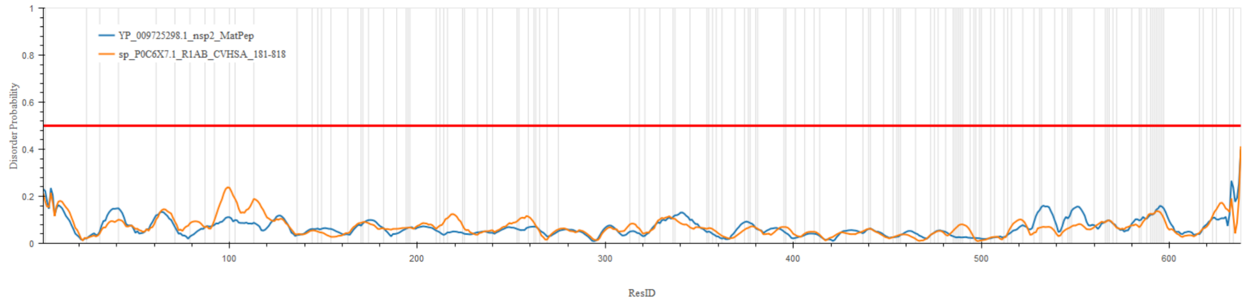

B

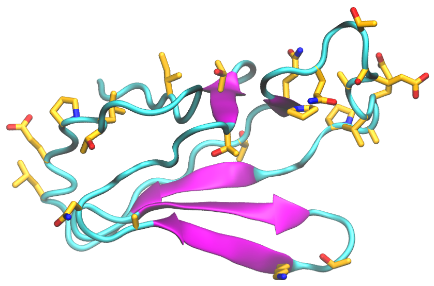

Fig. S5. Non-conservative substitutions in nsp2. A) Profile of disordered/structured regions of SARS-CoV-2 nsp2, predicted using DisEMBL (Linding et al. 2003), using the *hot loop* definition. The red line indicates the 50% threshold for disorder, comparing nsp2 SARS-CoV (*yellow* line), to nps2 SARS-CoV-2 (*blue* line). The grey vertical lines indicate the location of the non-conserved substitutions in SARS-CoV nsp2 relative to SARS-CoV-2 nsp2. B) *Ab initio* model of the C-terminal domain of SARS-CoV-2 nsp2 (a.a. 556-633). Non-conservative substitutions relative to SARS-CoV are depicted in *orange*.

Structural analysis and comparison with SARS-CoV nsp2 - Structure information about nsp2 is scarce. SARS-CoV-2 nsp2 is 68% identical to SARS-CoV nsp2. Among the substitutions, 130 are non-conservative and highly concentrated in the C-terminal end of the protein (Fig. S9A). An *ab initio* model was generated for this region (556-633, Fig. S9B). Fewer mutations appear at the N-terminal side, and a highly conserved region appears in the middle of the protein sequence.

Nonstructural protein 3 (nsp3) - Papain-like proteinase

The nonstructural protein 3 (nsp3) consists of multiple domains. One of these acts as a phosphatase and its catalytic domain is conserved, sharing homology with the Ymx7 protein in yeast, AF1521 in the archaea (*Archeoglobus fulgidus*), and Er58 in *E. coli*. (Saikatendu et al. 2005). The protein comprises at least six domains: 1) an N-terminal ubiquitin-like (Ubl1) domain followed by Glu-rich acidic domain; 2) an X domain with a predicted Appr-100-p processing activity; 3) a SUD domain (SARS-specific unique domain); 4) a peptidase C-16 domain that contains the papain-like protease (abbreviated PLnc); 5) a transmembrane domain; and 6) the Y domain.

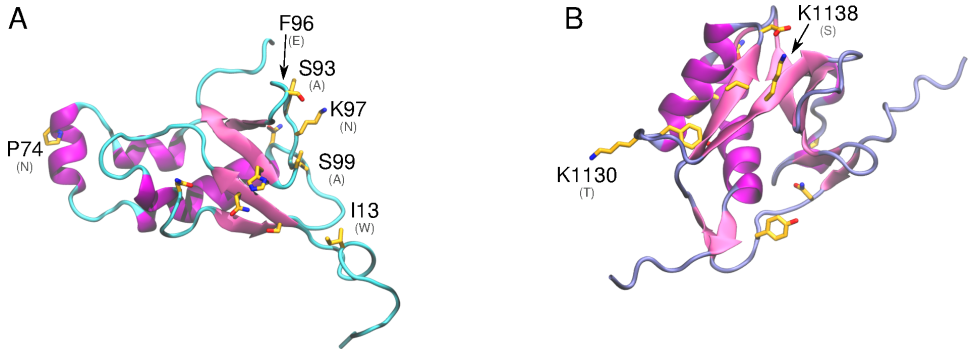

Fig. S6. Non-conservative substitutions in nsp3. A) Local modeling-based predicted structures of the N-terminal ubiquitin-like domain, and (B) the nucleic acid-binding domain of SARS-CoV-2 nsp3. Non-conservative substitutions relative to SARS-CoV are depicted in *orange*. These models were built based on PDB id: 2gri and 2k87.

Structural analysis and comparison with SARS-CoV nsp3 - Ub1 binds the nucleocapsid (N) protein and the interaction involves acidic residues of Ubl1 helix α2 and the serine and arginine-rich region of the N protein (Lei et al. 2018). Ub1 also binds to the 5' untranslated region of coronavirus RNA. Ub1 has significant structural homology with Ras effector proteins, and it is thought that Ub1 may interact with and modulate the activity of Ras in the host. This interaction potentially affects growth and cell cycle cascades and could be a potential mechanism for cell mortality (Serrano et al. 2007). Significantly, there are differences in residue identity between SARS-CoV and SARS-CoV-2 in residues 41 to 63, 83 to 87, 88 to 94, 95 to 98 of the Ub1 region (Fig. S6A). These regions correspond to structural homology with Ras effectors and these residue changes may affect interactions with host Ras and contribute to pathogenicity divergence (Serrano et al. 2007).

The Glu-rich acidic region is also known as the hypervariable region (HVR). It is intrinsically disordered and its interaction partners are not entirely clear. One study by yeast-2-hybrid demonstrates interaction with nsp6, but a GST pull-down assay also revealed interactions with nsp8, nsp9, and three regions of nsp3 itself (nucleic-acid binding domain, betacoronavirus-specific marker domain, and transmembrane region 1) (Lei et al. 2018). This region is significantly elongated, relative to the SARS-CoV counterpart, with 16 additional amino acids, including several potential sites of post-translational modification.

The X domain binds ADP-ribose, the structure of this complex was recently solved for SARS-CoV-2 (PDB id 6w02), and there are several studies and structures available in the Protein Data Bank of homolog complexes (PDB id: 5hol, 5dus, and 2fav). ADP-ribosylation is a type of post-translational modification (PTM), which may be implicated in inhibiting the immune response of the host through PTM of proteins related to the expression of interleukin-6 and interferon-beta (Lei et al. 2018). A GST-pulldown assay showed interaction of the X domain of nsp3 with nsp12. Several non-conservative substitutions occur in this domain relative to SARS-CoV, but those are located in superficial regions and do not significantly affect protein conformation (RMSD 1.5 Å, relative to 2fav). The papain-like protease domain (papain-like protease 2; PL2pro) (Lei et al. 2018) contains two ubiquitin-binding sites. It is implicated in suppression of the host immune response, but its targets and, more generally, which immune-related signal transduction cascades are affected is unclear. The substitution Gln^{977}Lys in this domain likely intensifies its interaction with ISG15 by forming a salt bridge with Glu^{127}, suggesting an important mechanism for variable virulence, as discussed in the main text (Fig. 6).

Finally, at the nucleic acid-binding domain (1089-1203), two substitutions relative to SARS-CoV add positive residues to the protein surface, Thr^{1130}Lys and Ser^{1138}Lys, and that may enhance the interaction with the phosphate groups from nucleic acids (Fig. S6B).

Nonstructural protein 4 (nsp4)

Working in coordination with nsp3 and nonstructural protein 6 (nsp6), the nonstructural protein 4 (nsp4) of SARS-CoV is essential for membrane rearrangements during viral replication (Angelini et al. 2013; Sakai et al. 2017). Data suggest that the coexpression of nsp3 with nsp4 results in host membrane rearrangement and the formation of double-membrane vesicles and convoluted membranes (Hagemeijer et al. 2014). Nsp4 in mouse hepatitis virus, another coronavirus, is a glycosylated protein and is demonstrated to be involved in virally induced membrane rearrangement, replication complex assembly, and assembly of double-membrane vesicles (Oostra et al. 2007; Sparks et al. 2007; Clementz et al. 2008; Gadlage et al. 2010; Beachboard et al. 2013). Aberrant double-membrane vesicles and impaired RNA replication can be observed when mouse hepatitis virus nsp4 is lacking a glycosylation site (Gadlage et al. 2010; Beachboard et al. 2015). Prevention of interaction between nsp4 and nsp3 eliminated viral replication (Sakai et al. 2017).

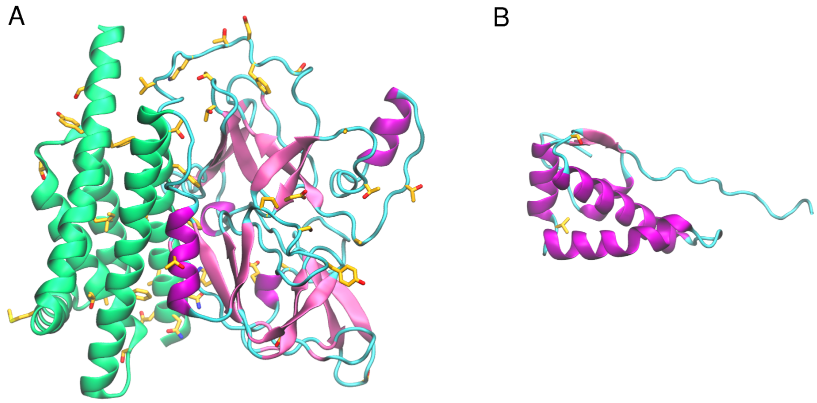

Fig. S7. **Non-conservative substitutions in nsp4.** A) *Ab initio* model of region 1-402 of SARS-CoV-2 nsp4. B) Fragment-based predicted structure of the conserved C-terminal domain of SARS-

CoV-2. Transmembrane regions are shown in *green*, soluble domains in *pink* and non-conservative substitutions relative to SARS-CoV nsp4 in *orange*.

Structural analysis and comparison with SARS-CoV nsp4 - Nsp4 of SARS-CoV is a nonstructural protein derived from the replicase polyprotein and is thought to be a tetra spanning transmembrane protein (Oostra et al. 2007). Disruption in glycosylation sites within the luminal loop between transmembrane domains 1 and 2 give rise to aberrant double-membrane vesicles (Gadlage et al. 2010). Key amino acid residues His^{120} and Phe^{121} of nsp4 of SARS-CoV, which are conserved in SARS-CoV-2, are essential for interaction and binding of nsp4 to nsp3 as well as viral propagation (Sakai et al. 2017).

Structural information about nsp4 is scarce. A low-resolution model was generated for the full nsp4 using the *ab initio* protocol (Fig. S7A). The soluble C-terminal domain is solved for a homologue (3vcb, MHV nsp4), enabling the generation of a higher resolution model for this region using the fragment-based modeling protocol (Fig. S7B). Despite the likely inaccurate orientation of domains in the full protein model, the predicted secondary structures are consistent with the expected positions of the transmembrane and soluble domains. This model is a useful resource for general structural analysis.

The sequence of SARS-CoV-2 nsp4 is 80% identical to SARS-CoV nsp4. Among the non-conservative substitutions, the predicted model reveals the substitution of four cysteines in the predicted TM helices by the bulky amino acids, Trp^{6}, Phe^{7}, Phe^{24}, and Phe^{390}. These substitutions may affect the arrangement and packing of transmembrane segments. Several substitutions are predicted to occur on the surface of the modeled soluble region spanning amino acids 33-275. The cytoplasmic C-terminal domain is highly conserved.

Nonstructural protein 6 (nsp6)

Upon infection, coronaviruses create signature membrane rearrangements (e.g. DMVs) so that the viral RNA replication complex may anchor to host machinery (den Boon and Ahlquist 2010). Nsp6, along with nsp3 and nsp4, plays a critical role in this membrane rearrangement (Angelini et al. 2013). In SARS-CoV, nsp6 is known to activate autophagy (Cottam et al. 2011) by inducing perinuclear vesicles localized around the microtubule organization center (Angelini et al. 2013).

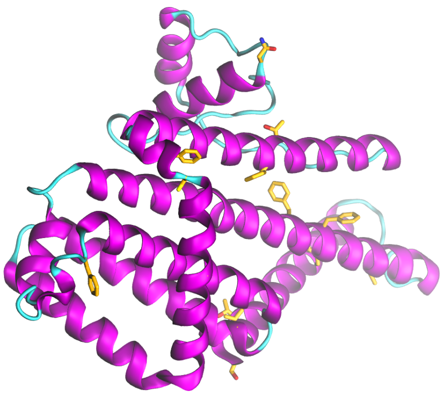

Fig. S8. Non-conservative substitutions in nsp6. *Ab initio* predicted structure of SARS-CoV-2 nsp6. Non-conservative substitutions relative to SARS-CoV nsp6 are depicted in *orange*.

Structural analysis and comparison with SARS-CoV nsp6 - SARS-CoV-2 nsp6 is 87% identical to SARS-CoV nsp6. Non-conservative mutations are located on the protein surface (Fig. S8).

Replication complex (nonstructural proteins 7, 8 and 12)

After infection, the virus assembles a multi-subunit RNA-synthesis complex consisting of several nonstructural proteins, namely nsp7, nsp8, nsp9, nsp10, and 12 (Smith and Denison 2013). This complex is responsible for the replication and transcription of the viral genome. The complex of nsp12, nsp7, and nsp8 (total 160 kDa) constitutes the minimum set of non-structural proteins required for nucleotide polymerization (Kirchdoerfer and Ward 2019). Nsp12 is the RNA-dependent RNA polymerase (RdRp), nsp7 forms a hexadecameric complex with nsp8 and, together, these may act as a processivity clamp for the RNA polymerase (Zhai et al. 2005). In the replication complex, there are two well-defined regions of nsp8: nsp8(I), forming the heterodimer with nsp7, and nsp8(II), binding to nsp12 (Kirchdoerfer and Ward 2019).

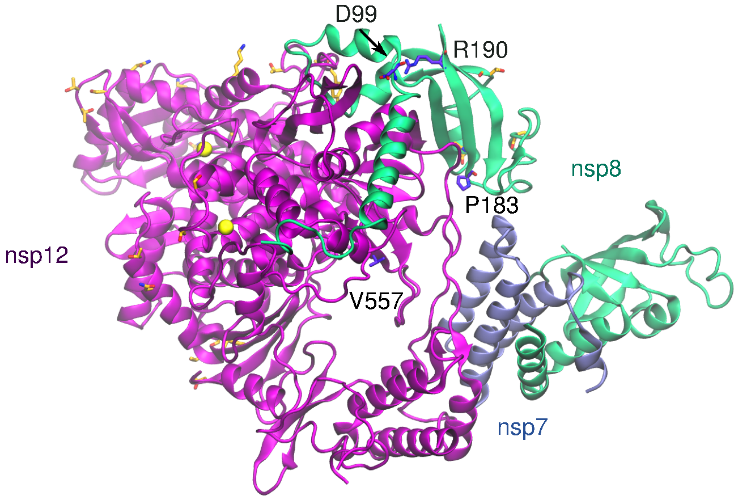

Fig. S9. Non-conservative substitutions in the replicase complex (nsp7-nsp8-nsp12, PDB id 7btf) (Gao et al. 2020). Non-conservative substitutions are depicted in *orange*. Some key functional residues identified are depicted in *purple*. Zinc ions are represented as *yellow* spheres.

Structural analysis and comparison with SARS-CoV nsp7-nsp8-nsp12 complex- A study conducted by Kirchdoerfer and Ward (2019), showed that the polymerase domain of SARS-CoV nsp12 (a.a 398-919) consists of the fingers domain (a.a 398–581, 628–687), palm domain (a.a. 582–627, 688–815) and a thumb domain (a.a. 816–919). The Nidovirus-unique N-terminal extension (aka NiRAN) is situated between a.a. 1–397. There are two metal (Zn) binding sites in nsp12: The first is in the NiRAN extension and is coordinated by residues His^{295}, Cys^{301}, Cys^{306}, and Cys^{310}. The second site is in the fingers domain and is coordinated by Cys^{487}, His^{642}, Cys^{645}, and Cys^{646}. The binding site for the nsp7-nsp8 heterodimer overlaps with the conserved regions of polymerase

functional domains (fingers (a.a 398–581, 628–687) and thumb domains (a.a. 816–919). The binding site between nsp8(II) and nsp12 is on the N-terminal region (77–126) of nsp8(II).

Several studies have pointed to functional residues in nsp7, 8, and 12 sequences. The study conducted by Lehmann et al. (2015), shows conserved sequence motifs in the NiRAN domain named A_N, B_N, and C_N. These motifs are conserved throughout all members of the order *Nidovirales*, of which CoVs are members (Lehmann et al. 2015). Another example is a covalent modification of the Lys^{73} residue in nsp12 (using GTP or UTP) that reduced viral growth and recovery in the equine arterivirus (Lehmann et al. 2015). *In vitro* polymerase activity assays further enabled the identification of other key functional sites, Lys^{7}, His^{36}, and Asn^{37}, which, when replaced by alanine, are associated with decreased polymerase activity (Subissi et al. 2014). Four other mutations in nsp8 were detrimental to polymerase activity namely, Pro^{183}Ala, Asp^{99}Ala, Pro^{116}Ala, and Arg^{190}Ala. These mutations are associated with a defective fold of nsp8 and disruption of nsp8(II)-nsp12 binding (Subissi et al. 2014). The nucleoside analog GS-5734 is capable of impairing CoV RNA synthesis by targeting the viral RNA synthesis machinery. The side chain in a motif of the fingers domain in nsp12 is involved in GS-5734 interaction through the Val^{557} residue (Agostini et al. 2018; C.J. Gordon et al. 2020). All of the mentioned residues are fully conserved in SARS-CoV-2 and all of the non-conservative substitutions are located in the complex surface, mostly in nsp12 (Fig. S9). Several of these substitutions involve residues that are potential sites for post-translational modifications (e.g., cysteines, serines, threonines, asparagines and tyrosines), indicating a variation in post-translational patterns relative to the SARS-CoV RNA polymerase complex.

Nonstructural protein 9 (nsp9)

The nonstructural protein 9 (nsp9) is cleaved from the viral polyproteins by the $3CL^{pro}$ protease (Sutton et al. 2004; Smith and Denison 2013). SARS-CoV nsp9 is able to form dimers that bind either ssDNA and ssRNA (Egloff et al. 2004) and is thought to protect the coronavirus genome from degradation during replication (Sutton et al. 2004; Ponnusamy et al. 2008). Deletion of nsp9 in the mouse β-coronavirus (hepatitis virus, MHV), impairs viral RNA synthesis and viral infection, where dimerization is particularly important (Deming et al. 2007; Miknis et al. 2009).

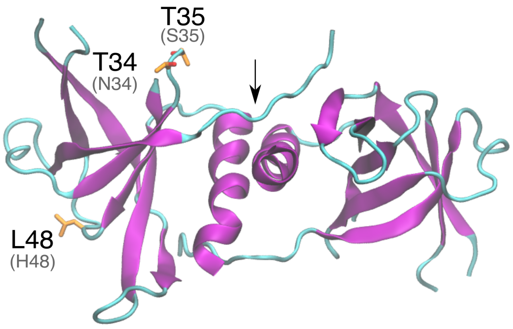

Fig. S10. Non-conservative substitutions in nsp9. Local modeling-based predicted structure of the SARS-CoV-2 nsp9 dimer. Arrow indicates the dimer interface and mutations are highlighted in *orange***.** PDB id 1uw7 was used as template.

Structural analysis and comparison with SARS-CoV nsp9 - Overall, the structure of nsp9 is very well conserved among β-coronaviruses (Sutton et al. 2004; Zeng et al. 2018). In SARS-CoV, nsp9 consists of a small globular protein (113 amino acid residues) with seven antiparallel β-sheets and one α-helix (Sutton et al. 2004). Parallel dimers occur between two nsp9 monomers interacting in their C-terminal α-helixes and their N-fingers (Zeng et al. 2018). In SARS-CoV, Gly^{100} and Gly^{104} residues are shown to be in the core of the dimer interface (Sutton et al. 2004). Mutation of these conserved glycines in the nsp9 α-helix impairs ssDNA binding, suggesting that dimerization is essential for nucleic acid binding (Zeng et al. 2018). Site-specific mutagenesis studies in the alpha-coronavirus Porcine Epidemic Diarrhea (PEDV) nsp9 identified other key residues for dimerization. The substitutions Lys^{10}Ala, Arg^{68}Ala, Lys^{69}Ala, and Arg^{106}Ala decreases binding affinity to form the dimer 7·2-fold relative to wild-type nsp9, while Tyr^{82}Ala enhance dimer stability, by an 8·0-fold increase in binding affinity (Zeng et al. 2018). Additionally, Zeng et al. (2018), studied Porcine Epidemic Diarrhea Virus (PEDV) nsp9 residues involved in ssDNA binding activity and they identified five mutations that affected this process, namely, Lys^{10}Ala, Arg^{68}Ala, Lys^{69}Ala, Arg^{106}Ala, and Tyr^{82}Ala. The equivalents in SARS-CoV-2 identified from a sequence alignment are Arg^{10}Ala, Arg^{74}Ala, Arg^{111}Ala, and Tyr^{87}Ala, which are conserved in SARS-CoV.

Because SARS-CoV and SARS-CoV-2 sequences are highly conserved (sequence identity is 97%), much structural information from SARS-CoV is transferable to SARS-CoV-2. The three substitutions in SARS-CoV-2 nsp2 relative to SARS-CoV, Asn^{34}Thr, Ser^{35}Thr, and His^{48}Leu, are located on the nsp9 surface, distant from the dimerization region (Fig. S10). Particularly, substitution Asn^{34}Thr can result in an additional phosphorylation site, but phosphorylation of nsp9 has not yet been reported. As Thr^{34} and Thr^{35} are close to a potential ubiquitination site (Lys^{36}), we hypothesize that their phosphorylation may prevent nsp9 ubiquitination.

Nonstructural protein 10 (nsp10)

Nsp10 is one of sixteen nonstructural proteins of the SARS-CoV-2 proteome. It interacts with nsp14 and nsp16 to perform 3’-5’ exoribonuclease and 2'-O-methyltransferase activities, respectively (Bouvet et al. 2010; Wang et al. 2015). Disturbance in the interaction between nsp10 and nsp16 has been shown to be crippling to the virus, although still functional (Bouvet et al. 2014). Nsp14, nsp16, and nsp10 have been associated with the coronavirus wide replication and transcription complex (Sawicki et al. 2005; Sawicki et al. 2006). Crystal structures of SARS-CoV nsp10-nsp16 have been reported (Decroly et al. 2011).

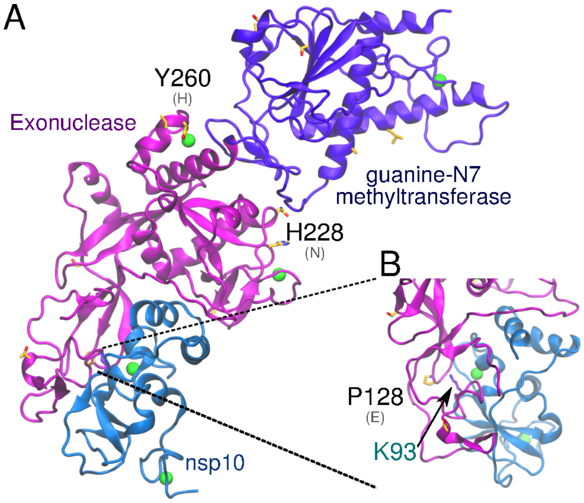

Fig. S11. Non-conservative substitutions in the nsp10-nsp14 complex. A) Local modeling-derived structure of SARS-CoV-2 nsp10-nsp14 complex. B) Closer view of residues Pro^{128} and Lys^{93} in nsp14 and nsp10 respectively. Non-conservative substitutions relative to SARS-CoV nsp10 and nsp14 are depicted in *orange*. Zinc ions are represented as *green* spheres. PDB 5c8s was used as the template to assemble the complex.

Structural analysis and comparison with SARS-CoV nsp9 - SARS-CoV and SARS-CoV-2 nsp10 proteins are highly conserved (identity 97%), with no non-conservative substitutions between them. These proteins are composed of 139 a.a. residues and fold forming two zinc fingers (Bouvet et al. 2014). This tight interaction with nsp14 (Fig. S11), involving multiple hydrogen bonds, salt bridges, and hydrophobic packing, suggests that nsp10 might be necessary to maintain the integrity of nsp14 exoribonuclease domain (Bouvet et al. 2012; Ma et al. 2015).

Nonstructural protein 11 (nsp11)

Nsp11 is a short protein only 13 a.a. in length and is translated from both polyproteins ORF1a and ORF1ab. In SARS-CoV nsp11 has been implicated in RNA synthesis (Su et al. 2006). SARS-CoV-2 nsp11 has 85% sequence identity to SARS-CoV nsp11, with two substitutions between them (Ser^5Gln and Thr^6Ser).

Nonstructural protein 13 (nsp13) - helicase

The nonstructural protein 13 (nsp13), encoded from ORF1ab as part of the pp1ab polyprotein, is involved in a number of functions, such as NTPase, dNTPase, RTpase, RNA helicase, and DNA helicase activity (Ivanov and Ziebuhr 2004). It also interacts with nsp12, which is thought to enhance its helicase activity (Ivanov and Ziebuhr 2004; Adedeji et al. 2012).

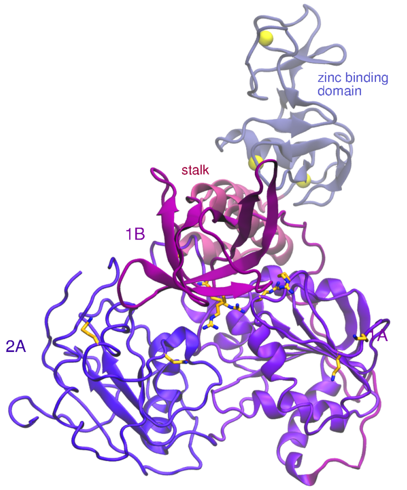

Fig. S12. Non-conservative substitutions in nsp13. Local modeling-based predicted structure of SARS-CoV-2 nsp13. Non-conservative substitutions relative to SARS-CoV nsp13 are depicted in *orange*. Mutations observed in SARS-CoV-2 nsp13 are depicted in *green*. Zinc ions are represented as *yellow* spheres. PDB 6jyt was used as the template.

Structural analysis and comparison with SARS-CoV nsp13 - The helicase nsp13 contains 5 domains, an N-terminal zinc-binding domain, stalk domain, RecA-like domain 1A, 1B, and RecA-like domain 2A, altogether consisting of 603 residues. It forms a triangular structure with the 2A and 1A as the base (Jia et al. 2019).

The zinc-binding domain consists of conserved cysteine residues, which have shown to be essential for the various enzymatic activities of nsp13 (Seybert et al. 2005). The stalk domain links the zinc-binding domain to the rest of the helicase, where the stalk domain has been argued to serve as an essential signal transduction mechanism (Jia et al. 2019). A single mutation in the stalk domain of SARS-CoV nsp13, Arg^{132}Pro, resulted in a dramatic decrease in viral infectivity *in vitro* (S. Fang et al. 2007). The essential nature of nsp13 in the viral replication cycle and its multifunctional nature have made it an attractive target for vaccine research (Wang et al. 2011); (Briguglio et al. 2011; Adedeji et al. 2014). SARS-CoV-2 nsp13 (Fig. S12) is fully conserved relative to SARS-CoV.

Nonstructural protein 14 (nsp14) - 3’ to 5’- exonuclease

The nonstructural protein nsp14 plays a critical role in coronaviruses replication and transcription. The exonuclease domain of nsp14 is imperative for replication fidelity within RNA viruses and has been shown to function as a proofreading exoribonuclease. Nsp14 associates with other nonstructural proteins from ORF1a and ORF1ab, including nsp7, nsp8, nsp12, and nsp10 (Eckerle et al. 2007; Eckerle et al. 2010; Denison et al. 2011; Subissi et al. 2014; Ma et al. 2015). The association with nsp10 provides the ability to excise mismatched nucleotides (Bouvet et al. 2012), and disruption of this heterodimer was shown to decrease replication fidelity (Smith et al. 2015).

Structural analysis and comparison with SARS-CoV nsp14 - Similar to SARS-CoV, SARS-CoV-2 nsp14 protein contains two catalytic domains: (1) The N-terminal exonuclease domain and (2) the C-terminal guanine-N7 methyltransferase domain. These domains, N-terminal and C-terminal,

span a.a. sequences 1-287 and 288-527, respectively. The exonuclease domain also has conserved residues Asp, Glu, Asp, and Asp (DEDD) that are key indicators of the DEDDh superfamily of DNA and RNA exonucleases (Zuo and Deutscher 2001; Smith and Denison 2013). The guanine-N7 methyltransferase domain has been shown to be necessary for viral mRNA capping (Ma et al. 2015), hiding viral mRNA from host mRNA degradation machinery.

Nsp14 contains two zinc-fingers within the N-terminal domain. The first is composed of Cys^{207}, Cys^{210}, Cys^{226}, and His^{229}, while the second is composed of His^{257}, Cys^{261}, His^{264}, and Cys^{279} [Ma et al. 2015]**.** Ala^{1}-Arg^{76} and Ala^{119}-Asp^{145} of nsp14 interact with nsp10. The third zinc-finger of nsp14 is within the C-terminal domain and is formed by Cys^{452}, Cys^{477}, Cys^{484}, and His^{487} (Ma et al. 2015).

SARS-CoV-2 nsp14 is 73% identical to the SARS-CoV counterpart. Among the 14 non-conservative substitutions, Glu^{128}Pro is the only substitution located close to the interface with nsp10 (Fig. S11B). The proximity with the positively charged Lys^{93} of nsp10 in the heterodimer model suggests that this substitution may slightly affect binding of nsp10-nsp14 (Fig. S11B). The substitutions His^{260}Tyr and Asn^{228}His may also be relevant as they are located in close proximity to the zinc-fingers in the N-terminal exonuclease domain (Fig. S11A).

Nonstructural protein 15 (nsp15) - endoRNAse

Nsp15 is a Nidoviral RNA uridylate-specific endoribonuclease (NendoU) and its C-terminal is a catalytic domain belonging to the EndoU family of enzymes (Johnson et al. 2010; Kim et al. 2020). EndoU enzymes have RNA endonuclease activity producing 2'-3' cyclic phosphodiester and 5'-hydroxyl termini (Ulferts and Ziebuhr 2011). A study in 2017 proposed that the NendoU activity interferes with the innate immune response (Deng et al. 2017). However, this finding was disputed in a 2019 study (Liu et al. 2019) that showed that the interference was independent of the endonuclease activity.

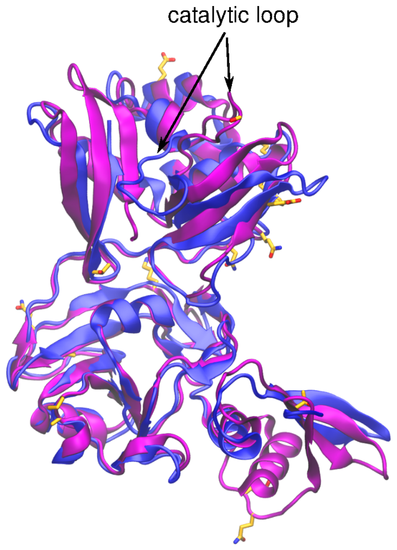

Fig. S13. Non-conservative substitutions in nsp15. Superimposed crystal structures of SARS-CoV (*blue,* PDB 2ozk) and SARS-CoV-2 (*purple,* PDB id 6vww) nsp15. Non-conservative residues in SARS-CoV-2 nsp15 relative to SARS-CoV are depicted in *orange*.

Structural analysis and comparison with SARS-CoV nsp15 - The nsp15 monomer (346 a.a.) forms hexamers. Each monomer consists of three domains, namely the N-terminal, middle domain, and catalytic NendoU domain at the C-terminal (Kim et al. 2020). Within SARS-CoV and SARS-CoV-2, nsp15 is very conserved (89% identity). However, the recently solved crystal structure of the nsp15 monomer shows a significant conformational variation in the region of the catalytic site. Although several non-conservative substitutions occur around this region, the conformation of the catalytic loop (a.a. 234-249) is known to greatly change upon protein oligomerization (Fig. S13).

Nonstructural protein 16 (nsp16) - 2'-O-ribose methyltransferase

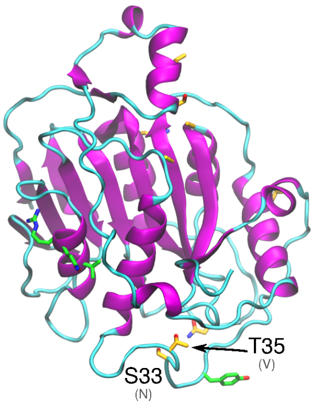

Fig. S14. Non-conservative substitutions in nsp16. Local modeling-derived structure of SARS-CoV-2 nsp16. Non-conservative substitutions relative to SARS-CoV nsp16 and depicted in *orange* and key functional residues are depicted in *green*. PDB id 3r24 was used as the template.

The nonstructural protein nsp16 is involved in capping of viral mRNA to protect it from host degradation, and it has been demonstrated that it has to be associated with nsp10 to be active. One potential method of ablating nsp16 activity is to disrupt the binding interface between nsp16 and nsp10. In SARS-CoV, the following interfacial mutations in nsp16 were shown to ablate nsp16 2'-O-methyltransferase activity: Ile^{40}Ala, Met^{41}Ala, Val^{44}Ala, Val^{78}Ala, Arg^{86}Ala, Val^{104}Gly, Leu^{244}Ala and Met^{247}Ala (Decroly et al. 2011). In a separate study, a double mutant (His^{83}Ala/Pro^{84}Ala) and a triple mutant (Tyr^{76}Ala/Cys^{77}Ala/Arg^{78}Ala) of nsp10 abolished SAM and m7GppA-RNA binding to nsp16 (Chen et al. 2011), further supporting the essential role of nsp10 in activating nsp16. In terms of the physicochemical interfacial properties that are essential for activating nsp16, it appears that certain hydrophobic interactions play a critical role and that enhancement of these may increase nsp16. This is exemplified by the Tyr^{96}Phe mutation in nsp10 that increases methyltransferase activity by increasing hydrophobic interaction between nsp10 and the hydrophobic pocket of nsp16, which includes the sidechain of Val^{84} and the mainchain portions of Gln^{87} and Arg^{86} (Chen et al. 2011). In SARS-CoV, residue Tyr^{30} in the RNA binding site of

nsp16, when mutated to either alanine or phenylalanine, abolished methlyltransferase activity, suggesting the critical role of this residue in RNA binding and, consequently, the enzymatic function (Decroly et al. 2011). All these key amino acids are conserved in SARS-CoV-2 nsp16, but non-conservative substitutions in the vicinity of Tyr^{30}, namely Asn^{33}Ser and Val^{35}Thr may affect RNA binding, as well as conservative substitutions (Glu^{32}Adp and Ile^{36}Leu) that may have a steric effect (Fig. S14).

ORF3a

ORF3a is the largest of all SARS-CoV accessory proteins and it is expressed in SARS-CoV infected cells, incorporated in viral particles (Shen et al. 2005). It is an integral transmembrane protein, localized mostly in the Golgi complex, cytoplasm, and cell surface (Yuan et al. 2005; Lu et al. 2006; Minakshi and Padhan 2014). ORF3a is able to form homodimers via disulfide bridges, which, in turn, form homotetramers through non-covalent interactions (Lu et al. 2006). The homotetramers have ion channel properties able to transport Na^+, K^+, and Ca^{+2} ions (Castaño-Rodriguez et al. 2018). ORF3a forms disulfide bonds with S protein via the cysteine-rich motif and a number of mutations in ORF3a correlate with mutations in the S protein in different SARS-CoV isolates, suggesting that its function is linked to the S protein (Zeng et al. 2004). Additionally, ORF3a and the E protein are both required for maximum viral growth (Castaño-Rodriguez et al. 2018).

Regarding pathogenic effects, ORF3a has been associated with inflammatory, NF-κB, IFN, apoptotic, cell cycle, and innate immunity responses (Kanzawa et al. 2006; Yuan et al. 2007; Padhan et al. 2008; Minakshi et al. 2009; Siu et al. 2019). From a clinical perspective, Obitsu et al. (2009) showed that ORF3a is able to induce osteoclastogenesis and, therefore, it is involved in the bone abnormalities observed individuals recovered from SARS. Further, ORF3a was reported to increase the expression of all subunits of fibrinogen, being associated with the development of pulmonary thrombosis upon SARS-CoV infection (Tan et al. 2005). Taken together, it is hypothesized that ORF3a has a role in the formation of blood clots in some patients.

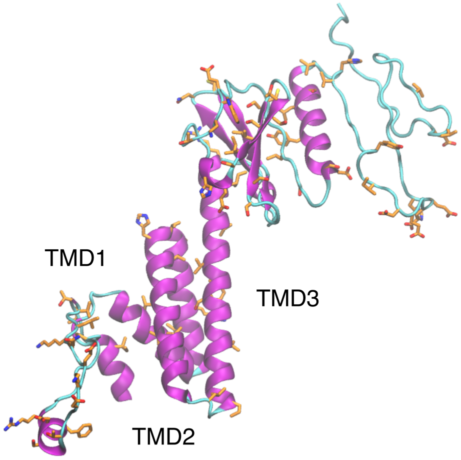

Fig. S15. Non-conservative substitutions in ORF3a. *Ab initio* predicted structure of SARS-CoV-2 ORF3a. Non-conservative substitutions relative to SARS-CoV ORF3a are depicted in *orange*.

Structural analysis and comparison with SARS-CoV ORF3a -

SARS-CoV-2 ORF3a is a protein of 275 residues with three transmembrane domains (TMD) and a C-terminal cytoplasmic domain (Fig. S15). The predicted transmembrane helices of SARS-CoV-2 ORF3a corresponds to residues 34 - 56 (TMD1), 77 - 99 (TMD2), and 103 - 125 (TMD3). SARS-CoV and SARS-CoV-2 ORF3a proteins share 72·36% identity. Several key functional residues, identified based on reports about SARS-CoV ORF3a, are conserved. For example, Tyr^{91} and His^{93} in TMD2, as well as Tyr^{109} in TMD3 are conserved, reinforcing the idea that these residues are important for ion channel activity (Castaño-Rodriguez et al. 2018). One mutation is found in the cysteine-rich motif Cys^{127}Leu (Leu-Trp-Leu-Cys-Trp-Lys-Cys). Residue Thr^{28}, known to be *O*-glycosylated in SARS-CoV ORF3a, is substituted by Phe^{28} in SARS-CoV-2. This substitution may affect ORF3a and S protein interaction, but it is likely that the other two cysteines are enough to establish the putative interaction (Zeng et al. 2004). The "tyrosine motif" (Tyr-Asn-Ser-Val) was fully conserved likely due to its importance for ORF3a function (Minakshi and Padhan 2014). In contrast, the substitution Glu^{171}Ser is found in the di-acidic domain (Ser-Gly-Asp) and may impair ORF3a apoptotic capacity (Chan et al. 2009). Three putative caveoline-1 binding sites are highly conserved, exhibiting only one non-conservative substitution, Tyr^{74}Ser (Padhan et al. 2007). Other non-conservative substitutions were identified in two potential sites of post-translational modification, Ser^{27}Asp and Thr^{28}Phe.

The sequence 125 to 200 is required for binding to the 5'-UTR of viral genomic RNA (Sharma et al. 2007). Several mutations are located in this region, including His^{152}Asn, Lys^{179}Ile, Lys^{181}Glu, Arg^{193}Trp, and His^{194}Glu. The subtraction/addition of charged or aromatic residues may have a significant impact on RNA binding affinity. However, this hypothesis requires empirical validation.

Envelope protein

The envelope (E) protein is one of the four structural proteins of coronaviruses. The other structural proteins are the membrane (M), nucleocapsid (N), and spike (S) proteins. The E protein is a type 1 transmembrane protein that is able to form pentamers by associating with other E proteins (Li et al. 2014). This pentamer forms a membrane pore that is able to transport ions (Li et al. 2014). The pore structure is called viroporin and it is present in many common viruses. The ion channel activity can be inhibited in SARS-CoV E protein by the drug HMA (Li et al. 2014). Only a few E proteins are present in each viral particle, but it is highly expressed in the host cells (Vennema et al. 1996; Nieto-Torres et al. 2011). The E protein has been proposed to initiate membrane curvature together with the M protein via the interaction between their C-terminal domains, but this mechanism remains largely unknown (Lim and Liu 2001; Schoeman and Fielding 2019). The M protein alone seems to not trigger a proper membrane curvature for virion production. The M protein alone can produce virions, but the absence of the E protein cripples virion production, morphology, and plaque shape (Vennema et al. 1996; Fischer et al. 1998; Lim and Liu 2001; Kuo and Masters 2003). The E protein localizes mainly in the ER and Golgi apparatus, where it participates in assembly, budding, and intracellular trafficking of newly formed virions (Vennema et al. 1996; Lim and Liu 2001).

The E protein has been shown to interact with other viral proteins. Tandem affinity purification assays established interaction between the S and the E proteins, but the mechanism of how this happens was not pursued further (Alvarez et al. 2010). This study also shows that the E protein

interacts with the nsp3 and they suggest that nsp3 mediates E protein ubiquitination. Besides, the E protein co-immunoprecipitates with the N protein, but the function of this interaction remains unclear (Maeda et al. 1999). Furthermore, a yeast two-hybrid system and an *in vitro* pull-down assay showed interactions between E proteins and ORF7a, but its importance is yet to be identified (Fielding et al. 2006; Pan et al. 2008). On the other hand, the E protein PDZ-Binding Motif (PBM) interacts with PDZ domains of host proteins. For instance, the interaction of the E protein and a protein associated with *Caenorhabditis elegans,* lin-7 protein 1 (PALS1), a PDZ-containing protein, disrupts tight junctions in the lungs to reach the alveolar wall and develops into a systemic infection (Teoh et al. 2010). Further, the interaction with syntenin caused it to concentrate in the cytoplasm, triggering an overexpression of inflammatory cytokines, which activates an exaggerated immune response, resulting in lung tissue damage, edema accumulation, and leading to acute respiratory distress syndrome (Jimenez-Guardeño et al. 2014). Interaction between the E protein and the Bcl-xL protein caused lymphopenia (Yang et al. 2005).

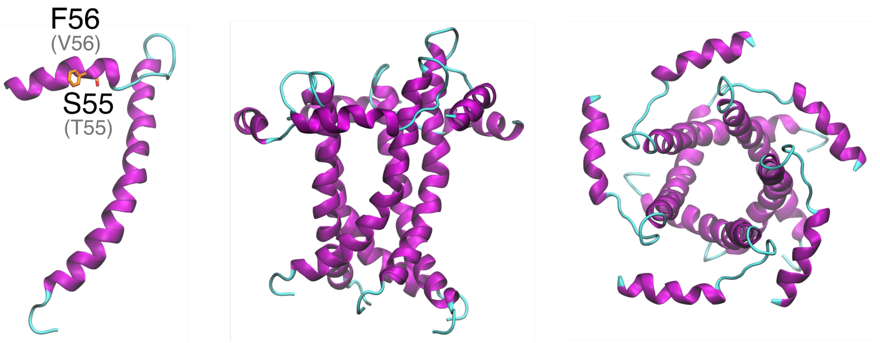

Fig. S16. Non-conservative substitutions in the envelope protein. Local modeling-based predicted model of E (based on PDB id 2mm4). E protein monomer and substitutions in the C-terminal relative to SARS-CoVs (*left*). E protein pentamer front (*middle*) and top views (*right*).

Structural analysis and comparison with SARS-CoV envelope protein - Several functionally important residues in E protein have been identified in SARS-CoV. For instance, there is a conserved proline residue in the C-terminal in the β-coil-β motif that, if mutated, changes localization of the E protein from the Golgi complex to the plasma membrane (Cohen et al. 2011; Li et al. 2014). Besides, the mutations Asn^{15}Ala and Val^{25}Phe inhibit ion channel activity of SARS-CoV E protein. After several passages through cell cultures, this function is restored by the addition of new mutations, suggesting that the E protein confers a selective advantage to the virus (Nieto-Torres et al. 2014). It is also important to note that the C-terminal of the E protein interacts with the C-terminal of the M protein in the cytoplasmic side, and this is required to virus envelope formation (Lim and Liu 2001). Since this protein is highly conserved relative to its SARS-CoV counterpart (96% identical), this information is likely applicable to SARS-CoV-2 E protein.

SARS-CoV-2 E protein has a predicted short 11 aa N-terminal tail, a 25 aa transmembrane region, and a 37 aa C-terminal cytoplasmic region, including PBM (DLLV). Four variations relative to SARS-CoV E protein were verified, all located in the C-terminal end (Fig. S16), including in one conservative substitution, two non-conservative substitutions (Val^{56}Phe, Glu^{69}Arg), and a deletion. Similar to SARS-CoV, SARS-CoV-2 E protein is likely to assemble as a pentamer to form a viroporin (Fig. S16), and the three C-terminal substitutions are likely exposed in the pentamer to

the cytoplasm, thereby they could be involved in modulating E protein interactions with other proteins.

Membrane protein

Membrane (M) proteins are the most abundant of all coronavirus structural proteins, and their presence and conformation determine the virion shape (Narayanan and Makino 2001; Kuo and Masters 2002). M protein has been shown to interact with viral proteins N, S, ORF3a and ORF7a (He, Dobie, et al. 2004; Huang et al. 2004; Tan et al. 2004; Fielding et al. 2006). The general structure of M proteins consists of a short N-terminal ectodomain, three transmembrane domains (TMD), and a C-terminal endodomain (Armstrong et al. 1984). M proteins may work as homodimers that can take on compact and long conformations (Neuman et al. 2011). The long conformation is associated with high S protein density and efficient virus budding, while compact conformation is associated with patchy and low S protein presence and inefficient budding (Neuman et al. 2011). Only M and E proteins are required to form virus-like particles (VLP), but the absence of S proteins makes VLPs appear larger, suggesting they may contribute to virus formation (Neuman et al. 2011). SARS-CoV M protein has been shown to interact with N protein in mammalian two-hybrid assays (He, Leeson, et al. 2004). Interaction of RNA and the M protein was found in mouse hepatitis virus (MHV) (Narayanan and Makino 2001).

The M protein has been demonstrated to be a pathogenic factor in coronaviruses. For instance, overexpression of M protein *in vitro* and *in vivo* induces apoptosis in human cell culture and *Drosophila*, respectively (Chan et al. 2007). Further, SARS-CoV M protein was found to interact with IKKβ, impairing NF-κB signaling and reducing the expression of cyclooxygenase-2 (COX-2) (X. Fang et al. 2007). COX-2 is responsible for the production of prostaglandins, which in turn trigger inflammatory responses. Furthermore, interferon (INF) is a group of antiviral proteins produced by cells in response to a viral infection. M protein impairs the production of type-I IFN by interfering with TRAF3-TANK-TBK1/IKKε complex formation, thus preventing IRF3 phosphorylation (Siu et al. 2009). Nonetheless, antigenic regions of M protein are detected and the production of IFN is triggered (Laude et al. 1992). These antigenic properties were observed in patients after a year of recovery from SARS-CoV infection, showing persistent IFNγ release from CD4+ and CD8+ lymphocytes when cocultured with M peptides (Yang et al. 2007). Thus, SARS-CoV infection triggered the production of memory cells, and M protein could be an interesting vaccine candidate.

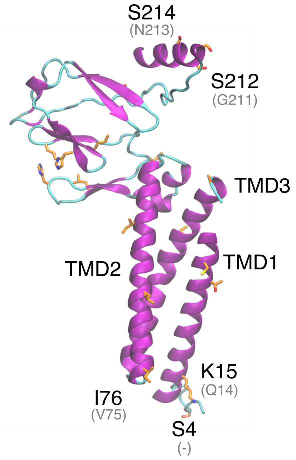

Fig. S17. Non-conservative substitutions in the membrane glycoprotein. *Ab initio* predicted structure of the SARS-CoV- 2 M protein. Conserved predicted regions and non-conservative substitutions (*black*) relative to SARS-CoV (*grey*) are depicted in *orange*.

Structural analysis and comparison with SARS-CoV membrane protein - Several key functional residues have been identified for the M protein. After several subculture passages in immortalized proximal tubular epithelial cells (PTEC), the SARS-CoV M protein generated a glutamic acid to alanine mutation at position 11 (Pacciarini et al. 2008). This mutation enhances virus replication and persistence in PTEC (Pacciarini et al. 2008). Tseng *et al*. (2013) identified other key residues in the SARS-CoV M protein with different functions in replication. The Leu-Leu motif (Leu^{218} and Leu^{219}) is required to incorporate the N protein into VLPs. Substitution Cys^{158}Ser reduces the secretion of the M protein, but not Cys^{63}Ser or Cys^{85}Ser, and Cys^{158} is involved in the interaction with the N protein. Non-conservative substitutions in the motif Ser-Trp-Trp-Ser-Phe-Asn-Pro-Glu reduced the production of VLPs. Conversely, substitutions of the aromatic residues in this motif by analogs did not affect the VLPs production, suggesting that the conservation of these aromatic amino acids is critical for M protein function.

The SARS-CoV-2 M protein is predicted to have three transmembrane domains, a cytoplasmic and a non-cytoplasmic domain. This is similar to the SARS-CoV M protein and other coronavirus M proteins (Armstrong et al. 1984; Hu et al. 2003). InterProScan (Jones et al. 2014) defined these regions as follows: non-cytoplasmic region (1 - 19), TMD1 (20 - 40), cytoplasmic region (41 - 51), TMD2 (52 - 73), non-cytoplasmic region (74 - 78), TMD3 (79 - 103) and cytoplasmic region (103 - 222). The SARS-CoV and SARS-CoV-2 M proteins are very similar, with 90·54% identity. Among 21 variations, 12 are non-conservative substitutions and one is an insertion. Within the substitutions, Gln^{15}Lys may affect antigenic properties of the M protein, by modifying its ectodomain (Fig. S17). Besides, Ser^{4} insertion is very close to a predicted N-glycosylation site at the N-terminal, and can constitute an additional glycosylation site. Modifications to this glycosylation site have been associated with induction of IFN production (Laude et al. 1992). Most of the non-conservative mutations occur in the cytoplasmic domain at the C-terminal domain.

ORF6

ORF6 is an auxiliary protein in SARS coronaviruses that is not required for virus replication (Yount et al. 2005; Huang et al. 2007). However, it can increase virus replication when expressed in a heterologous system or at low multiplicity of infection (Zhao et al. 2009). ORF6 localizes in the perinuclear and ER zones, associated with membranes and colocalized with M , S, and N structural proteins (Geng et al. 2005; Pewe et al. 2005). ORF6 is known to interact with the viral proteins ORF9b and nsp8 (Kumar et al. 2007; Calvo et al. 2012). Furthermore, ORF6 and nsp8 colocalize in cell culture, suggesting ORF6 may play a role in virus replication (Kumar et al. 2007).

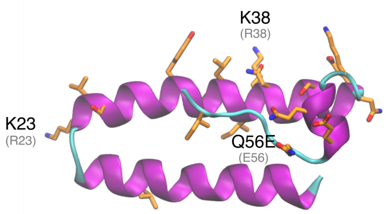

Fig. S18. Non-conservative substitutions in ORF6. *Ab initio* predicted structure of SARS-CoV-2 ORF6. Substitutions (*balck*) relative to SARS-CoV (*grey*) are shown in *orange*.

Structural analysis and comparison with SARS-CoV ORF6 - SARS-CoV ORF6 is a small protein of 63 amino acids and 7·53 kDa. Several important residues have been related to SARS-CoV ORF6 structure, function, and location. For instance, ORF6 sequence suggests a membrane association in residues 7 - 37 (Pewe et al. 2005). Even though some residues in this region are charged (Glu^{13}, Arg^{20} and Lys^{23}, in SARS-CoV-2 ORF6), this was enough for membrane association (Pewe et al. 2005).

ORF6 prevents import of the host signal transducer and activator of transcription 1-alpha/beta (STAT1) to the nucleus by tethering karyopherin subunit alpha-2 (KPNA2) and, therefore, KPNA1 to the ER and golgi apparatus membrane, thereby impairing the activation of INF-induced genes (Frieman et al. 2007). Tests with specific mutations in the C-terminal region of ORF6 revealed specific residues that affect disruption of KPNA2 ER and Golgi arrest in ORF6 transfected cells (Frieman et al. 2007). Further, the ORF6 C-terminal was shown to interact with Nmi protein, mediating Nmi ubiquitination and proteasome degradation, thus suppressing IFN signaling (Cheng et al. 2015). ORF6 N-terminal, in turn, was shown to induce membrane rearrangements typically observed in virus-infected cells and to have critical importance to prevent STAT1 translocation to the nucleus (Zhou et al. 2010).

SARS-CoV and SARS-CoV-2 ORF6 proteins are 68·85% identical. Most of the substitutions are located in the C-terminal helix (Fig. S18). Lysine substitutions Arg^{23}Lys, Arg^{38}Lys and Asn^{38}Lys in SARS-CoV-2 ORF6 suggest the introduction of new putative ubiquitination sites. ORF6 has been shown to be involved in proteasomal degradation of Nmi, but if ORF6 itself is regulated by the proteasome system is unknown.

Two studies pointed to residues 53 - 63 to be important for SARS-CoV ORF6 function (Frieman et al. 2007; Zhou et al. 2010). In this region, one non-conservative substitution (Glu^{56}Gln) and two deletions (Tyr^{62} and Pro^{63}) are verified.

ORF7a

ORF7a is an accessory protein of coronaviruses and it is not essential for viral replication *in vitro (Schaecher, Touchette, et al. 2007)*. ORF7a is a type I transmembrane protein, localized mainly in Golgi apparatus and in the cell surface (Nelson et al. 2005; Taylor et al. 2015). Besides, ORF7a colocalizes with calnexin (ER marker), showing that it is also localized at ER (Nelson et al. 2005).

ORF7a is an antagonist of bone marrow stromal antigen 2 (BST-2/CD317/tetherin) (Taylor et al. 2015). BST-2 is a pre-B-cell growth promoter that inhibits virus release by tethering budding virions to the host cell membrane (Sauter et al. 2010). A greater virion tethering to the cell membrane is observed when ORF7a is not present (Taylor et al. 2015). ORF7a is usually located in the Golgi apparatus and it relocates to the plasma membrane when BST-2 is expressed, colocalizing it (Taylor et al. 2015). ORF7a binds to BST-2 and reduces restriction activity of BTS2 by preventing its glycosylation (Taylor et al. 2015). Further *in vitro* experiments showed that ORF7a can induce apoptosis in a caspase dependent manner (Schaecher, Touchette, et al. 2007).

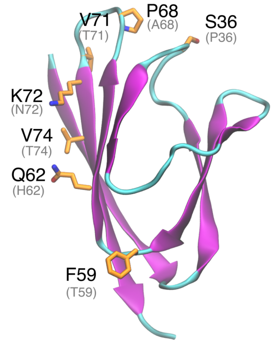

Fig. S19. Non-conservative substitutions in ORF7a. *Local modeling-based prediction of* SARS-CoV-2 ORF7a luminal domain (PDB id 1xak was used as template). Substitutions (*black*) relative to SARS-CoV (*grey*) are shown in *orange*.

Structural analysis and comparison with SARS-CoV ORF7a - SARS-CoV-2 ORF7a protein structure has a 15 amino acid (a.a.) N-terminal signal peptide, a 80-a.a. luminal domain, a 21-a.a. transmembrane domain, and a 5-a.a. cytoplasmic tail (Nelson et al. 2005). Residues Lys^{117}, Asn^{118} and Lys^{119} form a motif that has been described as being recognized by COPII vesicular system implicated in the transport of proteins from endoplasmic reticulum (ER) to Golgi and they are required to exit the ER (Bickford et al. 2004).

ORF7a structure is composed of seven antiparallel β-sheets that altogether make a β-sandwich (Fig. S19). The ORF7a is very conserved between SARS-CoV and SARS-CoV-2 with 85% sequence identity. A total of 18 variations are verified, with 11 non-conservative substitutions and a deletion. Substitutions in the luminal domain are located in the protein surface (Fig. S19). Therefore, the ORF7a fold is conserved, but these substitutions may affect ORF7a interaction with other viral or host proteins. Mostly conservative mutations are verified at ORF7a C-terminal (83 - 121), except by Ile^{111}Thr.

ORF7b

ORF7b is a small accessory protein expressed in SARS-CoV and SARS-CoV-2 infected cells with no sequence homology with other viral proteins (Schaecher, Mackenzie, et al. 2007). It is translated from a bicistronic open reading frame and encoded in the subgenomic RNA 7 and translated by ribosome leaky scanning (Schaecher, Mackenzie, et al. 2007). Like other accessory proteins, ORF7b is incorporated in viral particles and detected in purified virions (Schaecher, Mackenzie, et al. 2007). ORF7b is an integral transmembrane protein and it localizes in the cis- and trans- Golgi (Schaecher et al. 2008). Deletion of gene 7 does not affect replication kinetics in vitro, suggesting that ORF7b is not essential for virus replication (Pekosz et al. 2006).

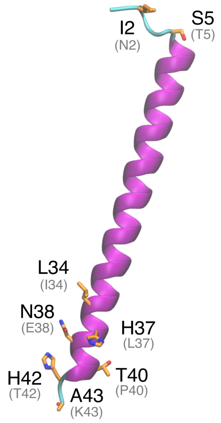

Fig. S20. Non-conservative substitutions in ORF7b. *Ab initio* predicted structure of SARS-CoV-2 ORF7b protein. Substitutions (*balck*) relative to SARS-CoV (*grey*) are shown in *orange*.

Structural analysis and comparison with SARS-CoV ORF7b - SARS-CoV ORF7b is a small protein of 44 amino acids with a transmembrane domain (TMD) of 22 residues (Schaecher et al. 2008). Mutations in the TMD affect ORF7b cellular localization(Schaecher et al. 2008). Alanine scanning experiments, identified that residues 13-15 and 19-22 are critical for ORF7b retention in the Golgi complex. SARS-CoV-2 ORF7b is 81% identical to SARS-CoV ORF7b. All substitutions are found in the terminals, meaning the TMD is fully conserved (Fig. S20).

ORF8

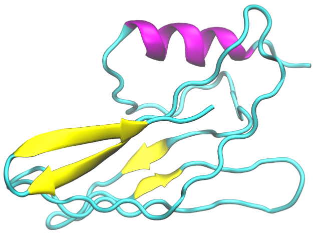

Fig. S21. ***Ab initio*** **predicted structure of SARS-CoV-2 ORF8.**

SARS-CoV-2 ORF8 is most similar to sequences from Bat-SARS-like coronavirus and a bat coronavirus RaTG13 (Hu et al. 2018). Remarkably, removal of a N-terminal 29 nucleotide sequence from SARS-CoV ORF8 in early tests showed a decrease in viral replication by up to 23-fold (Muth et al. 2018). This sequence was observed to be removed from the virus in human cases later in the outbreak along with additional and different mutations of ORF8 (Chinese SARS Molecular Epidemiology Consortium 2004a; Chiu et al. 2005). The exact purpose of this mutation and ORF8's function in regards to viral benefit within human hosts remains unknown. With the deletion, SARS-CoV encodes ORF8a and ORF8b, while SARS-CoV-2 ORF8 is intact (Guan et al. 2003; Chinese SARS Molecular Epidemiology Consortium 2004b; Lau et al. 2005; D.E. Gordon et al. 2020a). SARS-CoV-2 ORF8 (Fig. S21) is 127 a.a. long and 45% identical to SARS-CoV ORF8b.

Nucleocapsid protein

Nucleocapsid- Highly abundant in infected cells, SAR-CoV nucleocapsid (N) packages the viral RNA into a ribonucleocapsid and plays a key role in viral assembly (Chang et al. 2009). In SARS-CoV, it was demonstrated that the nucleocapsid self oligomerizes via a Ser-Arg rich region (a.a. 184-196) (He, Dobie, et al. 2004). It is the only structural protein that interacts with the replication/transcription complex (Verheije et al. 2010), binding via the same Ser-Arg rich region (Hurst et al. 2013).

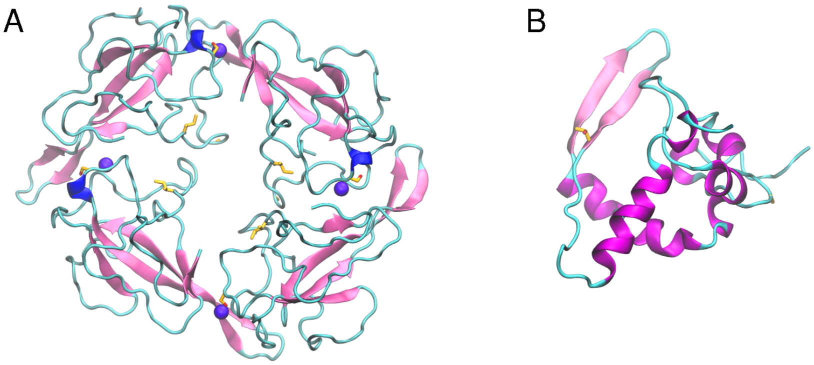

Fig. S22. Non-conservative substitutions in the nucleocapsid protein. A) Crystal structure of SARS-CoV-2 nucleocapsid RNA-binding domain (PDB id 6vyo). B) Local modeling-derived structure of SARS-CoV-2 nucleocapsid C-terminal (PDB id 2cjr was used as template). Non-conservative substitutions relative to SARS-CoV nucleocapsid are depicted in *orange*. Zinc ions are represented as *violet* spheres.

Structural analysis and comparison with SARS-CoV nucleocapsid - SARS-CoV-2 N protein is a 419 a.a. long protein and is 89% identical to SARS-CoV nucleocapsid. In SARS-CoV, a Ser-Arg rich motif (Ser-Ser-Arg-Ser-Ser-Ser-Arg-Ser-Arg-Gly-Asn-Ser-Arg) was found to be important for the oligomerization of N proteins (He, Dobie, et al. 2004). This motif is mostly conserved in SARS-CoV-2, except by the substitutions Gly^{192}Asn and Asn^{193}Ser. The latter may correspond to an additional phosphorylation site. Phosphorylation of this Ser/Arg-rich motif was described for

SARS-CoV N, and is reported to influence N antigenicity (Shin et al. 2007), nucleocytoplasmic shuttling (Surjit et al. 2005), and, the inhibition of the translocation of N to cytoplasmic stress granules (Peng et al. 2008). The RNA binding domain of the N protein is located at the N-terminal, within residues 47 and 180 of SARS-CoV-2. The structure of this domain, recently solved (PDB 6vyo) (figure S22A). Residues within the N-terminal of the SARS-CoV nucleocapsid, namely, Tyr^{87}, Tyr^{110}, Tyr^{112}, Y^{113}, Leu^{122}, and Ala^{13}, are hypothesized to play a role in ribonucleocapsid packaging (Chang et al. 2014). All of these residues are fully conserved in SARS-CoV-2 N. A long intrinsically disordered region follows these residues (181-246), and is predicted to fold upon binding (ANCHOR2 prediction (Mészáros et al. 2018)).

The oligomerization domain of nucleocapsids is situated in the C-terminal of SARS-CoV nucleocapsid. Residues Trp^{302}, Ile^{305}, Pro^{310}, Phe^{315}, and Phe^{316} are thought to be associated with highly hydrophobic interactions between two helices within the nucleocapsid (Chang et al. 2014), and they are fully conserved in SARS-CoV-2 N (figure S22B).

ORF9b

ORF9b is an accessory protein synthesized from an alternative reading frame in the N gene. This accessory protein is integrated in viral particles and therefore it can be considered a structural protein. This incorporation occurs in the presence of E and M proteins, suggesting a potential interaction among these proteins (Bouvet et al. 2010). ORF9b localizes in mitochondria outer membrane, cytoplasm, nucleus, endoplasmic reticulum and lipid vesicles (Meier et al. 2006; Moshynskyy et al. 2007; Calvo et al. 2012; Shi et al. 2014). At mitochondrial level, ORF9b promotes the ubiquitination and degradation of dynamin-like protein (DRP1), thereby causing mitochondria to show an elongated phenotype (Shi et al. 2014). Here, ORF9b also interacts with mitochondrial antiviral signaling protein (MAVS) and poly(rC) binding protein 2 (PCBP2) (Shi et al. 2014). Thus, PCBP2 facilitates the ubiquitination of MAVS by AIP4, suppressing the activation of IFN regulatory factors and NF-κB (Shi et al. 2014). Besides, interaction and colocalization between ORF9b and ORF6 have been suggested (Calvo et al. 2012), but the relevance of this interaction remains unknown.

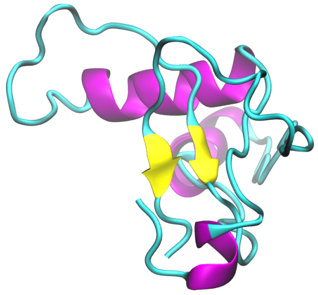

Fig. S23. Fragment-based predicted structure of SARS-CoV-2 ORF9b.

Structural analysis and comparison with SARS-CoV ORF9b - SARS-CoV ORF9b is a small protein of 98 residues length. Meier et al. (2006) showed that ORF9b forms a symmetric dimer where the monomer interactions resemble a handshake. Dimer assembling creates a hydrophobic tunnel that can accommodate a long fatty acid chain. The authors suggest that ORF9b could anchor

itself to a lipid membrane by internalizing one or more lipid tails. SARS-CoV-2 ORF9b (Fig. S23) shares high homology with SARS-CoV (72·45% protein identity).

ORF10

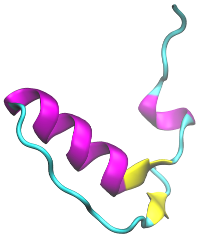

Fig. S24. ***Ab initio*** **predicted structure of ORF10.**

Within SARS-CoV-2, ORF10 suggested to interact with a Cullin 2 RING E3 ligase complex (D.E. Gordon et al. 2020a). Potentially, ORF10 might bind specifically to Cullin 2 ZYG11B complex and hijack this complex for ubiquitination and degradation.

Structural analysis of SARS-CoV-2 ORF10 - ORF10 of SARS-CoV-2 is the last predicted coding sequence upstream of the poly-A tail and is the shortest predicted coding sequence, composed of 38 a.a. ORF10 is predicted to harbor a long helix and a pair of ϐ-strands. It appears to be unique to SARS-CoV-2 (Fig. S24).

Supplementary References

Adedeji AO, Marchand B, Te Velthuis AJW, Snijder EJ, Weiss S, Eoff RL, Singh K, Sarafianos SG. 2012. Mechanism of nucleic acid unwinding by SARS-CoV helicase. *PLoS One* 7:e36521.

Adedeji AO, Singh K, Kassim A, Coleman CM, Elliott R, Weiss SR, Frieman MB, Sarafianos SG. 2014. Evaluation of SSYA10-001 as a replication inhibitor of severe acute respiratory syndrome, mouse hepatitis, and Middle East respiratory syndrome coronaviruses. *Antimicrob Agents Chemother.* 58:4894–4898.

Agostini ML, Andres EL, Sims AC, Graham RL, Sheahan TP, Lu X, Smith EC, Case JB, Feng JY, Jordan R, et al. 2018. Coronavirus Susceptibility to the Antiviral Remdesivir (GS-5734) Is Mediated by the Viral Polymerase and the Proofreading Exoribonuclease. *mBio* 9:e00221-18.

Agrawal T, Gupta GK, Agrawal DK. 2012. Vitamin D deficiency decreases the expression of VDR and prohibitin in the lungs of mice with allergic airway inflammation. *Exp Mol Pathol* 93:74–81.

Alvarez E, DeDiego ML, Nieto-Torres JL, Jiménez-Guardeño JM, Marcos-Villar L, Enjuanes L. 2010. The envelope protein of severe acute respiratory syndrome coronavirus interacts with the non-structural protein 3 and is ubiquitinated. *Virology* 402:281–291.

Angelini MM, Akhlaghpour M, Neuman BW, Buchmeier MJ. 2013. Severe acute respiratory syndrome coronavirus nonstructural proteins 3, 4, and 6 induce double-membrane vesicles. *MBio* 4:e00524-13.

Armstrong J, Niemann H, Smeekens S, Rottier P, Warren G. 1984. Sequence and topology of a model intracellular membrane protein, E1 glycoprotein, from a coronavirus. *Nature.* 308:751–752.

Beachboard DC, Anderson-Daniels JM, Denison MR. 2015. Mutations across murine hepatitis virus nsp4 alter virus fitness and membrane modifications*. J Virol*. 89:2080–2089.

Beachboard DC, Lu X, Baker SC, Denison MR. 2013. Murine hepatitis virus nsp4 N258T mutants are not temperature-sensitive. *Virology* 435:210–213.

Bickford LC, Mossessova E, Goldberg J. 2004. A structural view of the COPII vesicle coat. *Curr Opin Struct. Biol.* 14:147–153.

den Boon JA, Ahlquist P. 2010. Organelle-like membrane compartmentalization of positive-strand RNA virus replication factories. *Annu Rev Microbiol*. 64:241–256.

Bouvet M, Debarnot C, Imbert I, Selisko B, Snijder EJ, Canard B, Decroly E. 2010. In vitro reconstitution of SARS-coronavirus mRNA cap methylation. *PLoS Pathog*. 6:e1000863.

Bouvet M, Imbert I, Subissi L, Gluais L, Canard B, Decroly E. 2012. RNA 3'-end mismatch excision by the severe acute respiratory syndrome coronavirus nonstructural protein nsp10/nsp14 exoribonuclease complex. *Proc Natl Acad Sci U S A.* 109:9372–9377.

Bouvet M, Lugari A, Posthuma CC, Zevenhoven JC, Bernard S, Betzi S, Imbert I, Canard B, Guillemot J-C, Lécine P, et al. 2014. Coronavirus Nsp10, a critical co-factor for activation of multiple replicative enzymes. *J Biol Chem.* 289:25783–25796.

Briguglio I, Piras S, Corona P, Carta A. 2011. Inhibition of RNA Helicases of ssRNA Virus Belonging to Flaviviridae, Coronaviridae and Picornaviridae Families. *Int J Med Chem* 2011:1–22.

von Brunn A, Teepe C, Simpson JC, Pepperkok R, Friedel CC, Zimmer R, Roberts R, Baric R, Haas J. 2007. Analysis of intraviral protein-protein interactions of the SARS coronavirus ORFeome. *PLoS One* 2:e459.

Calvo E, DeDiego ML, García P, López JA, Pérez-Breña P, Falcón A. 2012. Severe acute respiratory syndrome coronavirus accessory proteins 6 and 9b interact in vivo. *Virus Res*. 169:282–288.

Castaño-Rodriguez C, Honrubia JM, Gutiérrez-Álvarez J, DeDiego ML, Nieto-Torres JL, Jimenez-Guardeño JM, Regla-Nava JA, Fernandez-Delgado R, Verdia-Báguena C, Queralt-Martín M, et al. 2018. Role of severe acute respiratory syndrome coronavirus viroporins E, 3a, and 8a in replication and pathogenesis. *mBio*. 9:e02325-17.

Chan C-M, Ma C-W, Chan W-Y, Chan HYE. 2007. The SARS-coronavirus membrane protein induces apoptosis through modulating the Akt survival pathway. *Arch Biochem Biophys*. 459:197–207.

Chan C-M, Tsoi H, Chan W-M, Zhai S, Wong C-O, Yao X, Chan W-Y, Tsui SK-W, Chan HYE. 2009. The ion channel activity of the SARS-coronavirus 3a protein is linked to its pro-apoptotic function. Int. J. Biochem. *Cell Biol*. 41:2232–2239.

Chang C-K, Hou M-H, Chang C-F, Hsiao C-D, Huang T-H. 2014. The SARS coronavirus nucleocapsid protein – Forms and functions. *Antiviral Res.* 103:39–50.

Chang C-K, Hsu Y-L, Chang Y-H, Chao F-A, Wu M-C, Huang Y-S, Hu C-K, Huang T-H. 2009. Multiple nucleic acid binding sites and intrinsic disorder of severe acute respiratory syndrome coronavirus nucleocapsid protein: implications for ribonucleocapsid protein packaging. *J Virol.* 83:2255–2264.

Cheng W, Chen S, Li R, Chen Y, Wang M, Guo D. 2015. Severe acute respiratory syndrome coronavirus protein 6 mediates ubiquitin-dependent proteosomal degradation of N-Myc (and STAT) interactor. *Virol Sin*. 30:153–161.

Chen Y, Su C, Ke M, Jin X, Xu L, Zhang Z, Wu A, Sun Y, Yang Z, Tien P, et al. 2011. Biochemical and structural insights into the mechanisms of SARS coronavirus RNA ribose 2'-O-methylation by nsp16/nsp10 protein complex. *PLoS Pathog*. 7:e1002294.

Chinese SARS Molecular Epidemiology Consortium. 2004a. Molecular evolution of the SARS coronavirus during the course of the SARS epidemic in China. *Science* 303:1666–1669.

Chinese SARS Molecular Epidemiology Consortium. 2004b. Molecular evolution of the SARS coronavirus during the course of the SARS epidemic in China. *Science* 303:1666–1669.

Chiu RWK, Chim SSC, Tong Y-K, Fung KSC, Chan PKS, Zhao G-P, Lo YMD. 2005. Tracing SARS-coronavirus variant with large genomic deletion. *Emerg Infect Dis.* 11:168–170.

Clementz MA, Kanjanahaluethai A, O'Brien TE, Baker SC. 2008. Mutation in murine coronavirus replication protein nsp4 alters assembly of double membrane vesicles. *Virology* 375:118–129.

Cohen JR, Lin LD, Machamer CE. 2011. Identification of a Golgi complex-targeting signal in the cytoplasmic tail of the severe acute respiratory syndrome coronavirus envelope protein. *J Virol.* 85:5794–5803.

Cornillez-Ty CT, Liao L, Yates JR, Kuhn P, Buchmeier MJ. 2009. Severe Acute Respiratory Syndrome Coronavirus Nonstructural Protein 2 Interacts with a Host Protein Complex Involved in Mitochondrial Biogenesis and Intracellular Signaling. *J Virol.* 83:10314–10318.

Cottam EM, Maier HJ, Manifava M, Vaux LC, Chandra-Schoenfelder P, Gerner W, Britton P, Ktistakis NT, Wileman T. 2011. Coronavirus nsp6 proteins generate autophagosomes from the endoplasmic reticulum via an omegasome intermediate. *Autophagy* 7:1335–1347.

Decroly E, Debarnot C, Ferron F, Bouvet M, Coutard B, Imbert I, Gluais L, Papageorgiou N, Sharff A, Bricogne G, et al. 2011. Crystal structure and functional analysis of the SARS-coronavirus RNA cap 2’-O-methyltransferase nsp10/nsp16 complex. *PLoS Pathog.* 7:e1002059.

Deming DJ, Graham RL, Denison MR, Baric RS. 2007. Processing of Open Reading Frame 1a Replicase Proteins nsp7 to nsp10 in Murine Hepatitis Virus Strain A59 Replication. J Virol 81:10280–10291.

Deng X, Hackbart M, Mettelman RC, O’Brien A, Mielech AM, Yi G, Kao CC, Baker SC. 2017. Coronavirus nonstructural protein 15 mediates evasion of dsRNA sensors and limits apoptosis in macrophages. *Proc Natl Acad Sci U S A*. 114:E4251–E4260.

Denison MR, Graham RL, Donaldson EF, Eckerle LD, Baric RS. 2011. Coronaviruses: an RNA proofreading machine regulates replication fidelity and diversity. *RNA Biol.* 8:270–279.

Donoghue M, Hsieh F, Baronas E, Godbout K, Gosselin M, Stagliano N, Donovan M, Woolf B, Robison K, Jeyaseelan R, et al. 2000. A novel angiotensin-converting enzyme–related carboxypeptidase (ACE2) converts angiotensin I to angiotensin 1-9. *Circulation Res* 87:e1-e9.

Eckerle LD, Becker MM, Halpin RA, Li K, Venter E, Lu X, Scherbakova S, Graham RL, Baric RS, Stockwell TB, et al. 2010. Infidelity of SARS-CoV Nsp14-exonuclease mutant virus replication is revealed by complete genome sequencing. *PLoS Pathog.* 6:e1000896.

Eckerle LD, Lu X, Sperry SM, Choi L, Denison MR. 2007. High fidelity of murine hepatitis virus replication is decreased in nsp14 exoribonuclease mutants. *J Virol.* 81:12135–12144.

Egloff M-P, -P. Egloff M, Ferron F, Campanacci V, Longhi S, Rancurel C, Dutartre H, Snijder EJ, Gorbalenya AE, Cambillau C, et al. 2004. The severe acute respiratory syndrome-coronavirus replicative protein nsp9 is a single-stranded RNA-binding subunit unique in the RNA virus world. *Proc Natl Acad of Sci USA* 101:3792–3796.

Fang S, Chen B, Tay FPL, Ng BS, Liu DX. 2007. An arginine-to-proline mutation in a domain with undefined functions within the helicase protein (Nsp13) is lethal to the coronavirus infectious bronchitis virus in cultured cells. *Virology* 358:136–147.

Fang X, Gao J, Zheng H, Li B, Kong L, Zhang Y, Wang W, Zeng Y, Ye L. 2007. The membrane protein of SARS-CoV suppresses NF-kappaB activation. *J Med Virol.* 79:1431–1439.

Fielding BC, Gunalan V, Tan THP, Chou C-F, Shen S, Khan S, Lim SG, Hong W, Tan Y-J. 2006. Severe acute respiratory syndrome coronavirus protein 7a interacts with hSGT. *Biochem Biophys Res Commun.* 343:1201–1208.

Fischer F, Stegen CF, Masters PS, Samsonoff WA. 1998. Analysis of Constructed E Gene Mutants of Mouse Hepatitis Virus Confirms a Pivotal Role for E Protein in Coronavirus Assembly. *J Virol.* 72:7885–7894.

Frieman M, Yount B, Heise M, Kopecky-Bromberg SA, Palese P, Baric RS. 2007. Severe acute respiratory syndrome coronavirus ORF6 antagonizes STAT1 function by sequestering nuclear import factors on the rough endoplasmic reticulum/Golgi membrane. *J Virol.* 81:9812–9824.

Gadlage MJ, Sparks JS, Beachboard DC, Cox RG, Doyle JD, Stobart CC, Denison MR. 2010. Murine hepatitis virus nonstructural protein 4 regulates virus-induced membrane modifications and replication complex function. *J Virol*. 84:280–290.

Gao Y, Yan L, Huang Y, Liu F, Zhao Y, Cao L, Wang T, Sun Q, Ming Z, Zhang L, et al. 2020. Structure of the RNA-dependent RNA polymerase from COVID-19 virus. *Science* 368:779-782.

Geng H, Liu Y-M, Chan W-S, Lo AW-I, Au DM-Y, Waye MM-Y, Ho Y-Y. 2005. The putative protein 6 of the severe acute respiratory syndrome-associated coronavirus: expression and functional characterization. *FEBS Lett.* 579:6763–6768.

Gordon CJ, Tchesnokov EP, Feng JY, Porter DP, Gotte M. 2020. The antiviral compound remdesivir potently inhibits RNA-dependent RNA polymerase from Middle East respiratory syndrome coronavirus. *J. Biol. Chem.* 295:4773-4779.

Gordon DE, Jang GM, Bouhaddou M, Xu J, Obernier K, White KM, O'Meara MJ, Rezelj VV, Guo JZ, Swaney DL, R, et al. 2020. A SARS-CoV-2 protein interaction map reveals drug targets and potential drug-repurposing. *Nature*. 583:459-468.

Graham RL, Sims AC, Brockway SM, Baric RS, Denison MR. 2005. The nsp2 replicase proteins of murine hepatitis virus and severe acute respiratory syndrome coronavirus are dispensable for viral replication. *J Virol.* 79:13399–13411.

Guan Y, Zheng BJ, He YQ, Liu XL, Zhuang ZX, Cheung CL, Luo SW, Li PH, Zhang LJ, Guan YJ, et al. 2003. Isolation and characterization of viruses related to the SARS coronavirus from animals in southern China. *Science* 302:276–278.

Hagemeijer MC, Monastyrska I, Griffith J, van der Sluijs P, Voortman J, van Bergen en Henegouwen PM, Vonk AM, Rottier PJM, Reggiori F, de Haan CAM. 2014. Membrane rearrangements mediated by coronavirus nonstructural proteins 3 and 4. *Virology* 458-459:125–135.

Hagemeijer MC, Verheije MH, Ulasli M, Shaltiël IA, de Vries LA, Reggiori F, Rottier PJM, de Haan CAM. 2010. Dynamics of coronavirus replication-transcription complexes. *J Virol.* 84:2134–2149.

Hamming I, Timens W, Bulthuis MLC, Lely AT, Navis GJ, van Goor H. 2004. Tissue distribution of ACE2 protein, the functional receptor for SARS coronavirus. A first step in understanding SARS pathogenesis. *J Pathol*. 203:631–637.

Harmer D, Gilbert M, Borman R, Clark KL. 2002. Quantitative mRNA expression profiling of ACE 2, a novel homologue of angiotensin converting enzyme. *FEBS Letters* 532:107–110.

He R, Dobie F, Ballantine M, Leeson A, Li Y, Bastien N, Cutts T, Andonov A, Cao J, Booth TF, et al. 2004. Analysis of multimerization of the SARS coronavirus nucleocapsid protein. *Biochem Biophys Res. Commun.* 316:476–483.

He R, Leeson A, Ballantine M, Andonov A, Baker L, Dobie F, Li Y, Bastien N, Feldmann H, Strocher U, et al. 2004. Characterization of protein–protein interactions between the nucleocapsid protein and membrane protein of the SARS coronavirus. *Virus Res.* 105:121–125.

Hernando-Rodríguez B, Artal-Sanz M. 2018. Mitochondrial Quality Control Mechanisms and the PHB (Prohibitin) Complex. *Cells* 7:238.

Huang C, Peters CJ, Makino S. 2007. Severe acute respiratory syndrome coronavirus accessory protein 6 is a virion-associated protein and is released from 6 protein-expressing cells. *J Virol.* 81:5423–5426.

Huang Y, Yang Z-Y, Kong W-P, Nabel GJ. 2004. Generation of synthetic severe acute respiratory syndrome coronavirus pseudoparticles: implications for assembly and vaccine production. *J Virol*. 78:12557–12565.

Hu D, Zhu C, Ai L, He T, Wang Y, Ye F, Yang L, Ding C, Zhu X, Lv R, et al. 2018. Genomic characterization and infectivity of a novel SARS-like coronavirus in Chinese bats. *Emerg Microbes Infect*. 7:154.

Hurst KR, Koetzner CA, Masters PS. 2013. Characterization of a critical interaction between the coronavirus nucleocapsid protein and nonstructural protein 3 of the viral replicase-transcriptase complex. *J Virol.* 87:9159–9172.

Hu Y, Wen J, Tang L, Zhang H, Zhang X, Li Y, Wang J, Han Y, Li G, Shi J, et al. 2003. The M Protein of SARS-CoV: Basic Structural and Immunological Properties. *GPB* 1:118–130.

Ivanov KA, Ziebuhr J. 2004. Human coronavirus 229E nonstructural protein 13: characterization of duplex-unwinding, nucleoside triphosphatase, and RNA 5'-triphosphatase activities. *J Virol.* 78:7833–7838.

Jia HP, Look DC, Shi L, Hickey M, Pewe L, Netland J, Farzan M, Wohlford-Lenane C, Perlman S, McCray PB Jr. 2005. ACE2 receptor expression and severe acute respiratory syndrome coronavirus infection depend on differentiation of human airway epithelia*. J Virol.* 79:14614–14621.

Jia Z, Yan L, Ren Z, Wu L, Wang J, Guo J, Zheng L, Ming Z, Zhang L, Lou Z, et al. 2019. Delicate structural coordination of the Severe Acute Respiratory Syndrome coronavirus Nsp13 upon ATP hydrolysis. *Nucleic Acids Res*. 47:6538–6550.

Jimenez-Guardeño JM, Nieto-Torres JL, DeDiego ML, Regla-Nava JA, Fernandez-Delgado R, Castaño-Rodriguez C, Enjuanes L. 2014. The PDZ-binding motif of severe acute respiratory syndrome coronavirus envelope protein is a determinant of viral pathogenesis. *PLoS Pathog*. 10:e1004320.

Johnson MA, Chatterjee A, Neuman BW, Wüthrich K. 2010. SARS Coronavirus Unique Domain: Three-Domain Molecular Architecture in Solution and RNA Binding. *J Mol Biol* 400:724–742.

Jones P, Binns D, Chang H-Y, Fraser M, Li W, McAnulla C, McWilliam H, Maslen J, Mitchell A, Nuka G, et al. 2014. InterProScan 5: genome-scale protein function classification. *Bioinformatics* 30:1236–1240.

Kanzawa N, Nishigaki K, Hayashi T, Ishii Y, Furukawa S, Niiro A, Yasui F, Kohara M, Morita K, Matsushima K, et al. 2006. Augmentation of chemokine production by severe acute respiratory syndrome coronavirus 3a/X1 and 7a/X4 proteins through NF-κB activation. *FEBS Letters* 580:6807–6812.

Kathiria AS, Butcher LD, Feagins LA, Souza RF, Boland CR, Theiss AL. 2012. Prohibitin 1 modulates mitochondrial stress-related autophagy in human colonic epithelial cells. *PLoS One* 7:e31231.

Kim Y, Jedrzejczak R, Maltseva NI, Endres M, Godzik A, Michalska K, Joachimiak A. 2020. Crystal structure of Nsp15 endoribonuclease NendoU from SARS-CoV-2. *Protein Sci.* 29:1596-1605.

Kirchdoerfer RN, Ward AB. 2019. Structure of the SARS-CoV nsp12 polymerase bound to nsp7 and nsp8 co-factors. *Nat Commun.* 10:2342.

Knoops K, Kikkert M, van den Worm SHE, Zevenhoven-Dobbe JC, van der Meer Y, Koster AJ, Mommaas AM, Snijder EJ. 2008. SARS-coronavirus replication is supported by a reticulovesicular network of modified endoplasmic reticulum. *PLoS Biol*. 6:e226.

Kumar P, Gunalan V, Liu B, Chow VTK, Druce J, Birch C, Catton M, Fielding BC, Tan Y-J, Lal SK. 2007. The nonstructural protein 8 (nsp8) of the SARS coronavirus interacts with its ORF6 accessory protein. *Virology* 366:293–303.

Kuo L, Masters PS. 2002. Genetic evidence for a structural interaction between the carboxy termini of the membrane and nucleocapsid proteins of mouse hepatitis virus. *J Virol.* 76:4987–4999.

Kuo L, Masters PS. 2003. The Small Envelope Protein E Is Not Essential for Murine Coronavirus Replication. *J Virol.* 77:4597–4608.

Laude H, Gelfi J, Lavenant L, Charley B. 1992. Single amino acid changes in the viral glycoprotein M affect induction of alpha interferon by the coronavirus transmissible gastroenteritis virus. *J Virol.* 66:743–749.

Lau SKP, Woo PCY, Li KSM, Huang Y, Tsoi H-W, Wong BHL, Wong SSY, Leung S-Y, Chan K-H, Yuen K-Y. 2005. Severe acute respiratory syndrome coronavirus-like virus in Chinese horseshoe bats. *Proc Natl Acad Sci U S A.* 102:14040–14045.

Lehmann KC, Gulyaeva A, Zevenhoven-Dobbe JC, Janssen GMC, Ruben M, Overkleeft HS, van Veelen PA, Samborskiy DV, Kravchenko AA, Leontovich AM, et al. 2015. Discovery of an essential nucleotidylating activity associated with a newly delineated conserved domain in the RNA polymerase-containing protein of all nidoviruses. *Nucleic Acids Res*. 43:8416–8434.

Lei J, Kusov Y, Hilgenfeld R. 2018. Nsp3 of coronaviruses: Structures and functions of a large multi-domain protein. *Antiviral Res*. 149:58–74.

Letko M, Marzi A, Munster V. 2020. Functional assessment of cell entry and receptor usage for SARS-CoV-2 and other lineage B betacoronaviruses. *Nat Microbiol* 5:562–569.

Lim KP, Liu DX. 2001. The missing link in coronavirus assembly. Retention of the avian coronavirus infectious bronchitis virus envelope protein in the pre-Golgi compartments and physical interaction between the envelope and membrane proteins. *J Biol Chem.* 276:17515–17523.

Linding R, Jensen LJ, Diella F, Bork P, Gibson TJ, Russell RB. 2003. Protein disorder prediction: implications for structural proteomics. *Structure* 11:1453–1459.

Liu X, Fang P, Fang L, Hong Y, Zhu X, Wang D, Peng G, Xiao S. 2019. Porcine deltacoronavirus nsp15 antagonizes interferon-β production independently of its endoribonuclease activity. *Mol Immunol.* 114:100–107.

Li W, Moore MJ, Vasilieva N, Sui J, Wong SK, Berne MA, Somasundaran M, Sullivan JL, Luzuriaga K, Greenough TC, et al. 2003. Angiotensin-converting enzyme 2 is a functional receptor for the SARS coronavirus. *Nature* 426:450–454.

Li Y, Surya W, Claudine S, Torres J. 2014. Structure of a conserved Golgi complex-targeting signal in coronavirus envelope proteins. *J Biol Chem*. 289:12535–12549.

Lu W, Zheng B-J, Xu K, Schwarz W, Du L, Wong CKL, Chen J, Duan S, Deubel V, Sun B. 2006. Severe acute respiratory syndrome-associated coronavirus 3a protein forms an ion channel and modulates virus release. *Proc Natl Acad Sci U S A.* 103:12540–12545.

Maeda J, Maeda A, Makino S. 1999. Release of Coronavirus E Protein in Membrane Vesicles from Virus-Infected Cells and E Protein-Expressing Cells. *Virology* 263:265–272.

Martínez L, Andreani R, Martínez JM. 2007. Convergent algorithms for protein structural alignment. *BMC Bioinformatics* 8:306.

Ma Y, Wu L, Shaw N, Gao Y, Wang J, Sun Y, Lou Z, Yan L, Zhang R, Rao Z. 2015. Structural basis and functional analysis of the SARS coronavirus nsp14-nsp10 complex. *Proc Natl Acad Sci U S A.* 112:9436–9441.

Meier C, Aricescu AR, Assenberg R, Aplin RT, Gilbert RJC, Grimes JM, Stuart DI. 2006. The crystal structure of ORF-9b, a lipid binding protein from the SARS coronavirus. *Structure* 14:1157–1165.

Mészáros B, Erdos G, Dosztányi Z. 2018. IUPred2A: context-dependent prediction of protein disorder as a function of redox state and protein binding. *Nucleic Acids Res.* 46:W329–W337.

Miknis ZJ, Donaldson EF, Umland TC, Rimmer RA, Baric RS, Schultz LW. 2009. Severe acute respiratory syndrome coronavirus nsp9 dimerization is essential for efficient viral growth. *J Virol*. 83:3007–3018.

Minakshi R, Padhan K. 2014. The YXXΦ motif within the severe acute respiratory syndrome coronavirus (SARS-CoV) 3a protein is crucial for its intracellular transport. Virol. J. 11:75.

Minakshi R, Padhan K, Rani M, Khan N, Ahmad F, Jameel S. 2009. The SARS Coronavirus 3a protein causes endoplasmic reticulum stress and induces ligand-independent downregulation of the type 1 interferon receptor. *PLoS One* 4:e8342.

Moshynskyy I, Viswanathan S, Vasilenko N, Lobanov V, Petric M, Babiuk LA, Zakhartchouk AN. 2007. Intracellular localization of the SARS coronavirus protein 9b: evidence of active export from the nucleus. *Virus Res.* 127:116–121.

Muth D, Corman VM, Roth H, Binger T, Dijkman R, Gottula LT, Gloza-Rausch F, Balboni A, Battilani M, Rihtarič D, et al. 2018. Attenuation of replication by a 29 nucleotide deletion in SARS-coronavirus acquired during the early stages of human-to-human transmission. *Sci Rep.* 8:15177.

Narayanan K, Makino S. 2001. Cooperation of an RNA Packaging Signal and a Viral Envelope Protein in Coronavirus RNA Packaging. *J Virology* 75:9059–9067.

Nelson CA, Pekosz A, Lee CA, Diamond MS, Fremont DH. 2005. Structure and intracellular targeting of the SARS-coronavirus Orf7a accessory protein. *Structure* 13:75–85.

Netherton CL, Wileman T. 2011. Virus factories, double membrane vesicles and viroplasm generated in animal cells. *Curr Opin Virol*. 1:381–387.

Neuman BW, Kiss G, Kunding AH, Bhella D, Baksh MF, Connelly S, Droese B, Klaus JP, Makino S, Sawicki SG, et al. 2011. A structural analysis of M protein in coronavirus assembly and morphology. *J Struct Biol.* 174:11–22.

Ng KHL. 2005. Pulmonary artery thrombosis in a patient with severe acute respiratory syndrome. *Postgrad Med J* 81:e3–e3.

Nieto-Torres JL, Dediego ML, Alvarez E, Jiménez-Guardeño JM, Regla-Nava JA, Llorente M, Kremer L, Shuo S, Enjuanes L. 2011. Subcellular location and topology of severe acute respiratory syndrome coronavirus envelope protein. *Virology* 415:69–82.

Nieto-Torres JL, DeDiego ML, Verdiá-Báguena C, Jimenez-Guardeño JM, Regla-Nava JA, Fernandez-Delgado R, Castaño-Rodriguez C, Alcaraz A, Torres J, Aguilella VM, et al. 2014. Severe acute respiratory

syndrome coronavirus envelope protein ion channel activity promotes virus fitness and pathogenesis. *PLoS Pathog.* 10:e1004077.

Nie Y, Wang P, Shi X, Wang G, Chen J, Zheng A, Wang W, Wang Z, Qu X, Luo M, et al. 2004. Highly infectious SARS-CoV pseudotyped virus reveals the cell tropism and its correlation with receptor expression. *Biochem Biophys Res Commun.* 321:994–1000.

Obitsu S, Ahmed N, Nishitsuji H, Hasegawa A, Nakahama K-I, Morita I, Nishigaki K, Hayashi T, Masuda T, Kannagi M. 2009. Potential enhancement of osteoclastogenesis by severe acute respiratory syndrome coronavirus 3a/X1 protein. *Arch Virol* 154:1457–1464.

Oostra M, te Lintelo EG, Deijs M, Verheije MH, Rottier PJM, de Haan CAM. 2007. Localization and membrane topology of coronavirus nonstructural protein 4: involvement of the early secretory pathway in replication. *J Virol*. 81:12323–12336.

Pacciarini F, Ghezzi S, Canducci F, Sims A, Sampaolo M, Ferioli E, Clementi M, Poli G, Conaldi PG, Baric R, et al. 2008. Persistent replication of severe acute respiratory syndrome coronavirus in human tubular kidney cells selects for adaptive mutations in the membrane protein. *J Virol*. 82:5137–5144.

Padhan K, Minakshi R, Towheed MAB, Jameel S. 2008. Severe acute respiratory syndrome coronavirus 3a protein activates the mitochondrial death pathway through p38 MAP kinase activation. *J Gen Virol.* 89:1960–1969.

Padhan K, Tanwar C, Hussain A, Hui PY, Lee MY, Cheung CY, Peiris JSM, Jameel S. 2007. Severe acute respiratory syndrome coronavirus Orf3a protein interacts with caveolin. *J Gen Virol*. 88:3067–3077.

Pan J ’an, Peng X, Gao Y, Li Z, Lu X, Chen Y, Ishaq M, Liu D, Dediego ML, Enjuanes L, et al. 2008. Genome-wide analysis of protein-protein interactions and involvement of viral proteins in SARS-CoV replication. *PLoS One* 3:e3299.

Pekosz A, Schaecher SR, Diamond MS, Fremont DH, Sims AC, Baric RS. 2006. Structure, expression, and intracellular localization of the SARS-CoV accessory proteins 7a and 7b. *Adv Exp Med Biol.* 581:115–120.

Pewe L, Zhou H, Netland J, Tangudu C, Olivares H, Shi L, Look D, Gallagher T, Perlman S. 2005. A severe acute respiratory syndrome-associated coronavirus-specific protein enhances virulence of an attenuated murine coronavirus. *J Virol*. 79:11335–11342.

Ponnusamy R, Moll R, Weimar T, Mesters JR, Hilgenfeld R. 2008. Variable oligomerization modes in coronavirus non-structural protein 9. *J Mol Biol*. 383:1081–1096.

Ren X, Glende J, Al-Falah M, de Vries V, Schwegmann-Wessels C, Qu X, Tan L, Tschernig T, Deng H, Naim HY, et al. 2006. Analysis of ACE2 in polarized epithelial cells: surface expression and function as receptor for severe acute respiratory syndrome-associated coronavirus. *J Gen Virol*. 87:1691–1695.

Saikatendu KS, Joseph JS, Subramanian V, Clayton T, Griffith M, Moy K, Velasquez J, Neuman BW, Buchmeier MJ, Stevens RC, et al. 2005. Structural Basis of Severe Acute Respiratory Syndrome Coronavirus ADP-Ribose-1″-Phosphate Dephosphorylation by a Conserved Domain of nsP3. *Structure* 13:1665–1675.

Sakai Y, Kawachi K, Terada Y, Omori H, Matsuura Y, Kamitani W. 2017. Two-amino acids change in the nsp4 of SARS coronavirus abolishes viral replication. *Virology* 510:165–174.

Sauter D, Specht A, Kirchhoff F. 2010. Tetherin: holding on and letting go. *Cell* 141:392–398.

Sawicki SG, Sawicki DL, Younker D, Meyer Y, Thiel V, Stokes H, Siddell SG. 2005. Functional and genetic analysis of coronavirus replicase-transcriptase proteins. *PLoS Pathog.* 1:e39.

Sawicki SG, Sawicki DL, Younker D, Meyer Y, Thiel V, Stokes H, Siddell SG. 2006. Correction: Functional and Genetic Analysis of Coronavirus Replicase-Transcriptase Proteins. *PLoS Pathogens* 2:e17.

Schaecher SR, Diamond MS, Pekosz A. 2008. The transmembrane domain of the severe acute respiratory syndrome coronavirus ORF7b protein is necessary and sufficient for its retention in the Golgi complex. *J Virol.* 82:9477–9491.

Schaecher SR, Mackenzie JM, Pekosz A. 2007. The ORF7b protein of severe acute respiratory syndrome coronavirus (SARS-CoV) is expressed in virus-infected cells and incorporated into SARS-CoV particles. *J Virol.* 81:718–731.

Schaecher SR, Touchette E, Schriewer J, Buller RM, Pekosz A. 2007. Severe acute respiratory syndrome coronavirus gene 7 products contribute to virus-induced apoptosis. *J Virol.* 81:11054–11068.

Schoeman D, Fielding BC. 2019. Coronavirus envelope protein: current knowledge. *Virol J.* 16:69.

Serrano P, Johnson MA, Almeida MS, Horst R, Herrmann T, Joseph JS, Neuman BW, Subramanian V, Saikatendu KS, Buchmeier MJ, et al. 2007. Nuclear magnetic resonance structure of the N-terminal domain of nonstructural protein 3 from the severe acute respiratory syndrome coronavirus. *J Virol.* 81:12049–12060.

Seybert A, Posthuma CC, van Dinten LC, Snijder EJ, Gorbalenya AE, Ziebuhr J. 2005. A complex zinc finger controls the enzymatic activities of nidovirus helicases. *J Virol.* 79:696–704.

Sharma K, Surjit M, Satija N, Liu B, Chow VTK, Lal SK. 2007. The 3a accessory protein of SARS coronavirus specifically interacts with the 5'UTR of its genomic RNA, Using a unique 75 amino acid interaction domain. *Biochemistry* 46:6488–6499.

Shen S, Lin P-S, Chao Y-C, Zhang A, Yang X, Lim SG, Hong W, Tan Y-J. 2005. The severe acute respiratory syndrome coronavirus 3a is a novel structural protein. *Biochem Biophys Res Commun.* 330:286–292.

Shi C-S, Qi H-Y, Boularan C, Huang N-N, Abu-Asab M, Shelhamer JH, Kehrl JH. 2014. SARS-coronavirus open reading frame-9b suppresses innate immunity by targeting mitochondria and the MAVS/TRAF3/TRAF6 signalosome. *J Immunol.* 193:3080–3089.

Sims AC, Baric RS, Yount B, Burkett SE, Collins PL, Pickles RJ. 2005. Severe acute respiratory syndrome coronavirus infection of human ciliated airway epithelia: role of ciliated cells in viral spread in the conducting airways of the lungs. *J Virol.* 79:15511–15524.

Siu K-L, Kok K-H, Ng M-HJ, Poon VKM, Yuen K-Y, Zheng B-J, Jin D-Y. 2009. Severe acute respiratory syndrome coronavirus M protein inhibits type I interferon production by impeding the formation of TRAF3.TANK.TBK1/IKKepsilon complex. *J Biol Chem.* 284:16202–16209.

Siu K-L, Yuen K-S, Castaño-Rodriguez C, Ye Z-W, Yeung M-L, Fung S-Y, Yuan S, Chan C-P, Yuen K-Y, Enjuanes L, et al. 2019. Severe acute respiratory syndrome coronavirus ORF3a protein activates the NLRP3 inflammasome by promoting TRAF3-dependent ubiquitination of ASC. *FASEB J.* 33:8865–8877.

Smith EC, Case JB, Blanc H, Isakov O, Shomron N, Vignuzzi M, Denison MR. 2015. Mutations in coronavirus nonstructural protein 10 decrease virus replication fidelity. *J Virol*. 89:6418–6426.

Smith EC, Denison MR. 2013. Coronaviruses as DNA wannabes: a new model for the regulation of RNA virus replication fidelity. *PLoS Pathog*. 9:e1003760.

Sparks JS, Lu X, Denison MR. 2007. Genetic analysis of Murine hepatitis virus nsp4 in virus replication. *J Virol*. 81:12554–12563.

Subissi L, Posthuma CC, Collet A, Zevenhoven-Dobbe JC, Gorbalenya AE, Decroly E, Snijder EJ, Canard B, Imbert I. 2014. One severe acute respiratory syndrome coronavirus protein complex integrates processive RNA polymerase and exonuclease activities. *Proc Natl Acad Sci U S A*. 111:E3900–E3909.

Su D, Lou Z, Sun F, Zhai Y, Yang H, Zhang R, Joachimiak A, Zhang XC, Bartlam M, Rao Z. 2006. Dodecamer structure of severe acute respiratory syndrome coronavirus nonstructural protein nsp10. *J Virol.* 80:7902–7908.

Sutton G, Fry E, Carter L, Sainsbury S, Walter T, Nettleship J, Berrow N, Owens R, Gilbert R, Davidson A, et al. 2004. The nsp9 replicase protein of SARS-coronavirus, structure and functional insights. *Structure* 12:341–353.

Tan Y-J, Teng E, Shen S, Tan THP, Goh P-Y, Fielding BC, Ooi E-E, Tan H-C, Lim SG, Hong W. 2004. A novel severe acute respiratory syndrome coronavirus protein, U274, is transported to the cell surface and undergoes endocytosis. *J Virol.* 78:6723–6734.

Tan Y-J, Tham P-Y, Chan DZL, Chou C-F, Shen S, Fielding BC, Tan THP, Lim SG, Hong W. 2005. The severe acute respiratory syndrome coronavirus 3a protein up-regulates expression of fibrinogen in lung epithelial cells. *J Virol.* 79:10083–10087.

Taylor JK, Coleman CM, Postel S, Sisk JM, Bernbaum JG, Venkataraman T, Sundberg EJ, Frieman MB. 2015. Severe acute respiratory syndrome coronavirus ORF7a inhibits bone marrow stromal antigen 2 virion tethering through a novel mechanism of glycosylation interference. *J Virol.* 89:11820–11833.

Teoh K-T, Siu Y-L, Chan W-L, Schlüter MA, Liu C-J, Malik Peiris JS, Bruzzone R, Margolis B, Nal B. 2010. The SARS coronavirus E protein interacts with PALS1 and alters tight junction formation and epithelial morphogenesis. Mol Biol Cell 21:3838–3852.

Tseng C-TK, Tseng J, Perrone L, Worthy M, Popov V, Peters CJ. 2005. Apical entry and release of severe acute respiratory syndrome-associated coronavirus in polarized Calu-3 lung epithelial cells. *J Virol.* 79:9470–9479.

Tseng Y-T, Chang C-H, Wang S-M, Huang K-J, Wang C-T. 2013. Identifying SARS-CoV membrane protein amino acid residues linked to virus-like particle assembly. *PLoS One* 8:e64013.

Ulferts R, Ziebuhr J. 2011. Nidovirus ribonucleases: Structures and functions in viral replication. *RNA Biol.* 8:295–304.

Vennema H, Godeke GJ, Rossen JW, Voorhout WF, Horzinek MC, Opstelten DJ, Rottier PJ. 1996. Nucleocapsid-independent assembly of coronavirus-like particles by co-expression of viral envelope protein genes. *EMBO J.* 15:2020–2028.

Verheije MH, Hagemeijer MC, Ulasli M, Reggiori F, Rottier PJM, Masters PS, de Haan CAM. 2010. The coronavirus nucleocapsid protein is dynamically associated with the replication-transcription complexes. *J Virol.* 84:11575–11579.

Wang Y, Sun Y, Wu A, Xu S, Pan R, Zeng C, Jin X, Ge X, Shi Z, Ahola T, et al. 2015. Coronavirus nsp10/nsp16 methyltransferase can be targeted by nsp10-derived peptide in vitro and in vivo to reduce replication and pathogenesis. *J Virol.* 89:8416–8427.

Wang Z, Huang J-D, Wong K-L, Wang P-G, Zhang H-J, Tanner JA, Spiga O, Bernini A, Zheng B-J, Niccolai N. 2011. On the mechanisms of bananin activity against severe acute respiratory syndrome coronavirus. *FEBS J.* 278:383–389.

Yang L, Peng H, Zhu Z, Li G, Huang Z, Zhao Z, Koup RA, Bailer RT, Wu C. 2007. Persistent memory CD4+ and CD8+ T-cell responses in recovered severe acute respiratory syndrome (SARS) patients to SARS coronavirus M antigen. *J Gen Virol.* 88:2740–2748.

Yang Y, Xiong Z, Zhang S, Yan Y, Nguyen J, Ng B, Lu H, Brendese J, Yang F, Wang H, et al. 2005. Bcl-xL inhibits T-cell apoptosis induced by expression of SARS coronavirus E protein in the absence of growth factors. *Biochem J* 392:135–143

Yount B, Roberts RS, Sims AC, Deming D, Frieman MB, Sparks J, Denison MR, Davis N, Baric RS. 2005. Severe acute respiratory syndrome coronavirus group-specific open reading frames encode nonessential functions for replication in cell cultures and mice. *J Virol.* 79:14909–14922.

Yuan X, Li J, Shan Y, Yang Z, Zhao Z, Chen B, Yao Z, Dong B, Wang S, Chen J, et al. 2005. Subcellular localization and membrane association of SARS-CoV 3a protein. *Virus Res.* 109:191–202.

Yuan X, Yao Z, Wu J, Zhou Y, Shan Y, Dong B, Zhao Z, Hua P, Chen J, Cong Y. 2007. G1 phase cell cycle arrest induced by SARS-CoV 3a protein via the cyclin D3/pRb pathway. *Am J Respir Cell Mol Biol* 37:9–19.

Zeng R, Yang R-F, Shi M-D, Jiang M-R, Xie Y-H, Ruan H-Q, Jiang X-S, Shi L, Zhou H, Zhang L, et al. 2004. Characterization of the 3a protein of SARS-associated coronavirus in infected vero E6 cells and SARS patients. *J Mol Biol.* 341:271–279.

Zeng Z, Deng F, Shi K, Ye G, Wang G, Fang L, Xiao S, Fu Z, Peng G. 2018. Dimerization of Coronavirus nsp9 with Diverse Modes Enhances Its Nucleic Acid Binding Affinity. *J Virol.* 92.

Zhai Y, Sun F, Li X, Pang H, Xu X, Bartlam M, Rao Z. 2005. Insights into SARS-CoV transcription and replication from the structure of the nsp7–nsp8 hexadecamer. *Nat Struct Mol Biol.* 12:980–986.

Zhao J, Falcón A, Zhou H, Netland J, Enjuanes L, Pérez Breña P, Perlman S. 2009. Severe acute respiratory syndrome coronavirus protein 6 is required for optimal replication. *J Virol.* 83:2368–2373.

Zheng W, Li Y, Zhang C, Pearce R, Mortuza SM, Zhang Y. 2019. Deep-learning contact-map guided protein structure prediction in CASP13. *Proteins* 87:1149–1164.

Zhou H, Ferraro D, Zhao J, Hussain S, Shao J, Trujillo J, Netland J, Gallagher T, Perlman S. 2010. The N-terminal region of severe acute respiratory syndrome coronavirus protein 6 induces membrane rearrangement and enhances virus replication. *J Virol.* 84:3542–3551.

Zuo Y, Deutscher MP. 2001. Exoribonuclease superfamilies: structural analysis and phylogenetic distribution. *Nucleic Acids Res*. 29:1017–1026.
